# Cathepsin B-Deficient Mice Resolve *Leishmania major* Inflammation Faster in a T Cell-Dependent Manner

**DOI:** 10.1371/journal.pntd.0004716

**Published:** 2016-05-16

**Authors:** Orhan Rasid, Véronique Mériaux, Erin M. Khan, Chloé Borde, Ioana S. Ciulean, Catherine Fitting, Bénédicte Manoury, Jean-Marc Cavaillon, Noëlle Doyen

**Affiliations:** 1 Institut Pasteur, Département Infection et Epidémiologie, Unité Cytokines & Inflammation, Paris, France; 2 Cantacuzino National Research Institute, Bucharest, Romania; 3 Institut Necker Enfants Malades, INSERM U1151-CNRS UMR 8253, Hôpital Necker-Enfants Malades, Paris, France; 4 Université Paris Descartes, Sorbonne Paris Cité, Faculté de Médecine, Paris, France; Queensland Institute of Medical Research, AUSTRALIA

## Abstract

A critical role for intracellular TLR9 has been described in recognition and host resistance to *Leishmania* parasites. As TLR9 requires endolysosomal proteolytic cleavage to achieve signaling functionality, we investigated the contribution of different proteases like asparagine endopeptidase (AEP) or cysteine protease cathepsins B (CatB), L (CatL) and S (CatS) to host resistance during *Leishmania major* (*L*. *major*) infection in C57BL/6 (WT) mice and whether they would impact on TLR9 signaling. Unlike TLR9^-/-^, which are more susceptible to infection, AEP^-/-^, CatL^-/-^ and CatS^-/-^ mice are as resistant to *L*. *major* infection as WT mice, suggesting that these proteases are not individually involved in TLR9 processing. Interestingly, we observed that CatB^-/-^ mice resolve *L*. *major* lesions significantly faster than WT mice, however we did not find evidence for an involvement of CatB on either TLR9-dependent or independent cytokine responses of dendritic cells and macrophages or in the innate immune response to *L*. *major* infection. We also found no difference in antigen presenting capacity. We observed a more precocious development of T helper 1 responses accompanied by a faster decline of inflammation, resulting in resolution of footpad inflammation, reduced IFNγ levels and decreased parasite burden. Adoptive transfer experiments into alymphoid RAG2^-/-^γc^-/-^ mice allowed us to identify CD3+ T cells as responsible for the immune advantage of CatB^-/-^ mice towards *L*. *major*. *In vitro* data confirmed the T cell intrinsic differences between CatB^-/-^ mice and WT. Our study brings forth a yet unappreciated role for CatB in regulating T cell responses during *L*. *major* infection.

## Author Summary

Cutaneous forms of leishmaniasis are characterized by lesions that progress over months or years and that often leave permanent scars. Toll like receptors play an important role in the recognition and initiation of immune responses, and the intracellular TLR9, a sensor of pathogen double-stranded DNA, plays a crucial role in host resistance to *Leishmania* parasites. To achieve functionality, proteolytic enzymes, like cathepsins B, L, or S or asparagine endopeptidase, must cleave TLR9. Using mice deficient for different cathepsins, we demonstrate that these cathepsins do not seem to be individually involved in TLR9 processing. Interestingly we observed that Cathepsin B-deficient mice were more resistant to *Leishmania major* infection, meaning they resolve lesions and reduce parasite burdens faster than wild-type C57BL/6 mice. We found that this resistance is based on adaptive rather than innate immunity, with a central role of Cathepsin B-deficient T cells that contribute to faster controls of *leishmania* probably by higher IFNγ production. Cathepsin B inhibitors were already shown to have favorable effect in leishmaniasis, but the mechanisms behind these effects remain unclear. Our study highlights a new role for cathepsin B at the T cell level and provides new clues to how targeting this molecule is beneficial for treating *leishmania* infections.

## Introduction

A protective immune response against intracellular protozoan parasites of the genus *Leishmania* is characterized by the development of IFNγ-producing T cells. This supports macrophages in the induction of anti-leishmanial effector functions, such as production of nitric oxide [1,2]. IL-12, a cytokine produced largely by antigen-presenting cells (APCs), such as dendritic cells (DCs), contributes to immunity against *Leishmania major* (*L*. *major)* by both polarizing and supporting T helper (Th) 1 responses [3]. The capacity of DCs to produce IL-12 is directly conditioned by the recognition of pathogen associated molecular patterns (PAMPs). This is achieved through a variety of receptors, of which Toll-like receptors (TLRs) are by far the best characterized [4,5]. A large body of knowledge has been accumulated on the recognition of *Leishmania* by different TLRs [6,7]. We, and others, have previously described a critical role for intracellular TLR9, a sensor of pathogen double-stranded DNA, in recognition and host resistance to *Leishmania* parasites [8–12]. TLR9 requires a proteolytic cleavage step inside the endolysosome to achieve signaling functionality. TLR9 maturation was proposed to be a multistep process requiring, among other molecules, the contribution of asparagine endopeptidase (AEP) and other cysteine proteases such as cathepsins B (CatB), L (CatL) or S (CatS) [13–16]. Although analysis of TLR9 processing and signaling supported a role for both cathepsins and AEP in macrophages and DCs, there is no consensus on their contribution to TLR9 maturation and its consequences on innate immunity. In *Leishmania* infection, despite the known importance of DCs in polarizing Th responses and the role of cysteine proteases in modulating DC functions, the role of these proteins remains poorly understood. The importance of the Th1/Th2 balance for protective immunity in leishmaniasis is clearly illustrated by the susceptibility of the prototypical Th2 BALB/c mouse strain as opposed to the resistance of Th1-prone C57BL/6 or DBA/2 mice [1].

Cathepsins have been the subject of a few studies during *Leishmania* infection and use of specific inhibitors has been shown to skew Th responses [17–19]. Inhibiting CatB was suggested to favor the development of protective Th1 responses in BALB/c but not in DBA/2, whereas inhibition of CatL exacerbated the disease in both BALB/c and DBA/2 mice [18,19]. Still, further research is needed to elucidate the mechanisms behind these effects. The role of AEP and CatS however, has not been investigated in *L*. *major* infection. We thus set out to investigate how AEP, CatB, CatL and CatS affect the immune response during *L*. *major* infection with the aim of assessing if TLR9-dependent responses are affected by these proteases. Comparing mice deficient in AEP, CatB, CatL and CatS, we observed that only CatB-deficient mice were more resistant to infection, meaning they resolve lesions and reduce parasite burdens faster than C57BL/6 (WT) mice. We went on to test the involvement of CatB in TLR9-dependent DC responses and found no impact of CatB. We performed a thorough analysis of the local immune responses to *L*. *major* and found no major differences in the innate immune response to infection between WT and CatB^-/-^ mice. We however found a clear difference in the Th1 response between the two mouse strains, observing a somewhat faster apparition and a consistently speedier decline of IFNγ levels in CatB^-/-^ mice. Having observed no differences in innate responses or antigen presenting capacity of DCs we investigated whether CatB might affect lymphocyte populations, as previous studies have suggested [20–23]. Using a series of adoptive transfer and *in vitro* experiments we found that CD3+ T are intrinsically different in CatB mice and can confer an immune advantage to recipient alymphoid mice upon transfer, as compared to their WT counterparts. To our knowledge this is the first study to identify a T cell-intrinsic role for CatB in *L*. *major* infection and thus setting the stage for a new direction of research into the role of cysteine proteases in *Leishmania* protozoan infections.

## Materials and Methods

### Ethics statement

Animals were housed in the Institut Pasteur animal facilities accredited by the French Ministry of Agriculture to perform experiments on mice in compliance of the French and European regulations on care and protection of the Laboratory Animals (EC Directive 86/609, French Law 2001–486 issued on June 6, 2001). The CETEA (Comité d'Ethique pour l'Expérimentation Animale—Ethics Committee for Animal Experimentation) “Paris Centre et Sud" reviewed and approved the animal care and use protocol under the approval number 2012–0059.

### Mice, parasites and reagents

Six to 8 week-old female C57BL/6 mice were purchased from Charles River Laboratories. TLR9^-/-^ mice, backcrossed to the C57BL/6 background for at least 10 generations were provided by S. Akira (Osaka University, Osaka, Japan). Mice deficient in asparagine endopeptidase, cathepsin B, cathepsin L, and cathepsin S, as previously described [24–27], and backcrossed to the C57BL/6 background for at least 10 generations were supplied by Dr. B. Manoury (Hospital Necker, Paris). These mice were bred in our facilities and housed under specific pathogen-free conditions. Genotypes of deficient mice were controlled by PCR on their genomic DNA. Rag2^-/-^γc^-/-^ mice were kindly provided by Dr. A. Galgano (IP Paris). Promastigotes of *L*. *major* (LV39) were propagated in vitro in M199 medium supplemented by 10% of foetal calf serum (FCS) (Biowest, France). Killed or live *L*. *major* promastigotes were in stationary phase; the killed *L*. *major* promastigotes were heat killed at 56°C for 30 min.

### Inoculation with *L*. *major*, lesion monitoring, and parasite burden

For infection, mice were inoculated subcutaneously (s.c.) into the footpads (FPs) with 3x10^6^ stationary phase *L*. *major* promastigotes. The size of the resulting lesions was assessed weekly by measuring the footpad size with a metric caliper (Kroeplin, Germany) and comparing it with the footpad thickness before infection. Each week after infection, blood was collected by retro-orbital bleeding for seric determinations, the draining lymph nodes and the footpads were harvested for cellular analysis and tissue parasite burden, determined by limiting dilution analysis as previously described [28].

### Cell culture and stimulation

Bone marrow derived dendritic cells (BMDCs) and bone marrow derived macrophages (BMDMs) were differentiated from bone marrow cells, obtained from the mice detailed above, as previously described [29]. In brief, cells were grown in complete RPMI 1640 with GlutaMAX (Gibco) with 10% FCS (Biowest), 1mM sodium pyruvate, HEPES 10mM, MEM non-essential amino acids, 2Mercaptoethanol 0,05mM and gentamicin 0,02mg/ml (all from Gibco). BMDCS and BMDMs were differentiated with GM-CSF from J558L cell line supernatants [30] or M-CSF from L929 fibroblast cell line supernatants, respectively. At day 8 and 7, respectively, BMDCs were 80% CD11c+CD11b+ and BMDMs were 95% CD11c-CD11b+. Total cells were harvested and cultured in 6-well plates (3x10^6^/well) and stimulated for 6 h with killed or live *L*. *major* promastigotes at a parasite/cell ratio of 5:1, or 250 ng/ml of CpG 1826 or 100ng/ml of conventional LPS (E. coli, O111:B4, Sigma). Cells were harvested at 6h after stimulation for RNA extraction. At 24h, cell-free supernatants were harvested for cytokine determination by specific ELISA.

### Preparation of footpads, lymph nodes or splenic cells

Cells were isolated from footpads, lymph nodes and spleens of naive or *L*. *major*-infected mice at various times after infection. The footpad tissue was cut into small pieces that were gently separated with a pestle and digested with liberase 1,5mg/ml in PBS at 37° for 1 h. The reaction was stopped with FCS 10% in RPMI. Then in a second step cells were separated from the remaining tissue by a digestion with collagenase type 4 (0.5 mg/ml) and DNase type I (40 ug/ml) (Boehringer-Mannheim) for 30 min stopped with PBS with 10% FCS. Cells were then washed, filtered through a 70μm cell strainer and used for FACS analysis. Single cell suspensions from draining popliteal lymph nodes or spleens were prepared by crushing organs through a 70μm cell strainer before staining for FACS analysis.

### Stimulation and proliferation of naïve purified T cells

CD3 or CD4 T cells were isolated from the spleens of C57BL/6 and CatB^-/-^ mice and washed in PBS. CD3 or CD4 isolation was achieved by specific negative enrichment kit (eBioscience and Miltenyi Biotec, respectively) according to manufacturer’s protocol. Purity of isolated cells was verified by flow cytometry and was always around 95%. To study the proliferation of CD4+ T cells, 2x10^6^/ml purified cells were labelled with carboxyfluorescein succinimidyl ester (CFSE) at the concentration of 5μM/ml in PBS without any trace of EDTA or protein at 37°C during 10 minutes. The reaction was stopped with culture medium with 10% FCS and the labeling was controlled by FACS. The cells were resuspended in complete RPMI medium at 2x10^5^/ml, anti-CD28 was added at (2,5 μg/ml) and the cells were plated on anti-CD3 or isotype-coated wells (5 μg/ml) with and incubated at 37°in CO_2_ 5% incubator for 72h. CFSE dilutions were analysed by FACS. Naïve CD3+ purified cells from WT and CatB^-/-^ were incubated at the concentration 2x10^5^/ml at 37°C in complete medium with phorbol 12-myristate 13-acetate (PMA) 200 ng/ml and 500 ng/ml of ionomycine. Supernatants were harvested at 24, 48, and 72h and cytokine production was assessed by specific ELISA.

### ELISA

IL-1β, IL-6, TNF, IL-10, CXCL1, CCL2 and CCL5 were quantified in cell culture or footpad supernatants using ELISA kits (BD Biosciences). Weight-matched footpad tissues were dilacerated and incubated in 400 μl of PBS with protease inhibitor, 4 hours at room temperature to allow cytokines diffusion before harvesting the supernatants.

All ELISA procedures were performed according to the manufacturer’s protocol.

### RNA extraction and quantitative RT-PCR

RNA from cells or from lymph-nodes and footpads tissue was extracted using respectively a microRNeasy extraction kit (Qiagen) or TRIzol Reagent (Thermo Fisher Scientific), according to the manufacturer’s protocols. RNA (2 μg) was reverse transcribed using (200 U) Moloney murine leukemia virus reverse transcriptase (SuperScript II, Invitrogen). Subsequent real time PCR was performed on Step One Plus (Applied Biosystems) using Taq polymerase (Taq-Man Universal PCR master mix, Applied biosystems). All PCR data values were normalized to the expression of the hypoxanthine phosphoribosyltransferase (HPRT) gene.

### Flow cytometry

Single cell suspensions from footpads, draining lymph nodes or spleens were surface stained for phenotypic analysis. Cells were incubated for 30 minutes with appropriate amounts of antibodies in the presence of *Fc* receptor-blocking agent (Fc block from BD Bioscience), after which cells were washed and stained with a viability dye (eBioscience). Antibodies used were directed against mouse CD45 (GL2), CD11c (HL3), CD40 (3/23), CD3 (145-2CII), CD4 (RM4-5), CD8 (53–6.7), CD25 (PC65), CD19 (ID3), CD11b (M1/70), Ly6G (1A8-Ly6G), Ly6C (HK1.4) and NK1.1 (PK136) from BD Biosciences and eBioscience. For intracellular staining, surface-labeled cells were fixed and permeabilized with the Inside Stain Kit from Miltenyi Biotec for IFNγ labelling with an anti-IFNγ (XMG1.2) or fixed and permeabilized with the transcription factor permeabilization kit from eBioscience and stained for foxp3 (MF23 from BD Biosciences). All controls were stained with the respective isotypes. Flow cytometric data were acquired on a MACSQuant device (Miltenyi Biotec) and analysed using FlowJo software (TreeStar).

### Measurement of *L*. *major* antigen specific seric immunoglobulins (IgGs)

Soluble leishmanial antigens (SLA) were prepared using 10^9^/ml stationary phase promastigotes of *L*. *major* as previously described [19]. The aliquots were stored at -80^0^ and thawed only one time. SLA (2ug/ml in PBS) were covalently coated on to 96 well plates (Nunc Maxisorp Roskilde, Denmark) for 2 hours at room temperature and overnight at 4°C. After blocking with 10% FCS in PBS, the wells were incubated with serum samples serially diluted in the blocking buffer for 2 hours at room temperature. After washing the plates were incubated for 90 min at room temperature with horseradish peroxidase (HRP) -Goat anti-mouse IgG (H+L) (Invitrogen) diluted at 1/5000 or -Goat anti-Mouse IgG1 diluted at 1/4000 or -Goat Anti-Mouse IgG2a diluted at 1/1000 (both from Human ads-HRP Southern Biotech). After washing with Tween 0,1%, BSA 1%, bound antibodies were revealed with TMB (TMB Substrate Reagent Set from BD) and the colorimetric reaction was read at 450nm.

### Reconstitution of Rag2^-/-^γc^-/-^ mice with splenocytes or purified CD3 + T

Spleen cells were isolated from C57BL/6 and CatB^-/-^ mice and washed in PBS. Cell preparation were either kept on ice or processed for CD3 cell isolation before adoptive transfer. CD3 isolation was achieved by negative enrichment kit (eBioscience) according to manufacturer’s protocol. Purity of isolated cells was verified by flow cytometry and was always >94%. Splenocytes or purified CD3 cells were resuspended at a concentration of 10^6^ cells/100 μl PBS and adoptively transferred by retro-orbital intravenous (i.v.) injection of 100 μl into recipient Rag2^-/-^γc^-/-^ mice. On day 7 after adoptive transfer, mice were inoculated s.c. into the footpads with 6x10^6^ stationary phase *L*. *major* promastigotes and lesions size was monitored as described above. The *in vivo* proliferation of T cells was done in Rag2^-/-^γc^-/-^ mice, 21 days after CD3 transfer as described above but without any infection. On day 21, each mouse was injected in intrapertoneal (i.p.) with 1 mg of 5-Bromo-2′-deoxyuridine (BrdU), 12h later a second injection of 1 mg BrdU was performed, and at 16h a third injection [31]. One hour after the last injection, spleens were harvested and BrdU labelling was performed according to manufacturer’s instructions using the BrdU flow kit from BD Pharmingen.

### *L*. *major* antigen presentation by BMDCs

Cells from draining lymph nodes of C57BL/6 or CatB^-/-^ mice were harvested 21 or 28 days after *L*. *major* inoculation. Lymph node single cells suspensions or purified CD4+ T cells, at a purity of around 95%, were obtained by negative selection with a CD4+ T cell isolation kit (Miltenyi Biotec) using manufacturer’s instructions, then used as stimulus target cells. BMDCs from C57BL/6 or CatB^-/-^ mice, 2,5x10^5^ were cultured in 24 well plates at 37°C and 5% CO_2_ using complete RPMI 1640 and stimulated with 1,25x10^6^ living or killed *L*. *majo*r promastigotes for 6h. After three washes in RPMI to remove free parasites, lymph node cells or purified CD4+ T cells were co-cultured with BMDCs at a ratio of 5:1 for 24h. For intracellular labelling of IFNγ, brefeldin A (5 μg/ml) was added for the last 4h of culture. Supernatants were harvested and frozen at -20°C for ELISA assays. Cells were used for intracellular detection of IFNγ by FACS, as described above. Controls included lymphocytes cultured alone, naïve splenic T cells, and co-cultures stimulated with PMA-ionomycine at 200 ng/ml and 500ng /ml, respectively.

### Statistical analysis

Statistical significance was tested using Prism 5.0 Software (GraphPad). student *t*-test and 2-way ANOVA comparisons with post-hoc Bonferroni tests were used as statistical tests. Error bars in all figures represent SEM, with the midlines representing the mean value in scatter plots.

## Results

### Unlike TLR9, asparagine endopeptidase and several cysteine proteases do not contribute to control *L*. *major*, while cathepsin B favors lesion persistence

**Fig 1:**
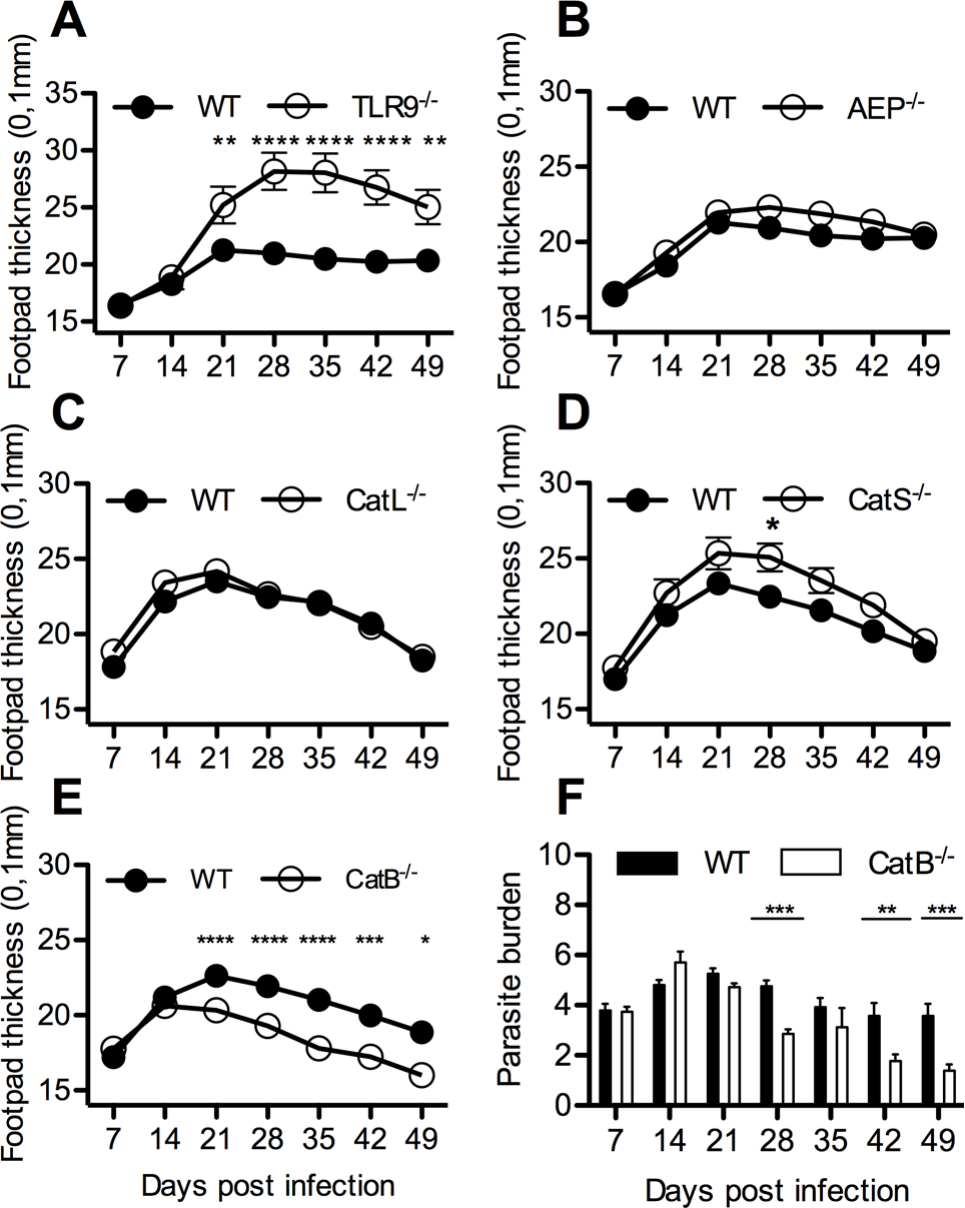
CatB^-/-^ mice resolve L. major subcutaneous infection faster than WT. WT, TLR9^-/-^, AEP^-/-^, CatL^-/-^, CatS^-/-^ and CatB^-/-^ were subcutaneously inoculated with 3x10^6^ stationary phase promastigotes of *L*. *major* in the footpads and experimental read-outs were measured weekly as indicated. (**A)** Mean footpad thickness (±SEM) of infected mice (WT–filled circle; TLR9^-/-^- empty circle). (**B)** Mean footpad thickness (±SEM) of infected mice (WT–filled circle; AEP^-/-^- empty circle). (**C)** Mean footpad thickness (±SEM) of infected mice (WT–filled circle; CatL^-/-^- empty circle). (**D)** Mean footpad thickness (±SEM) of infected mice (WT–filled circle; CatS^-/-^- empty circle) (**E)** Mean footpad thickness (±SEM) of infected mice (WT–filled circle; CatB^-/-^- empty circle). **(F)** Logarithm base 10 of limit dilution for parasite burden from footpads of infected mice (WT—filled bars; CatB^-/-^- empty bars), middle line represents mean value per group. Figures depict representative data pooled from at least 3 independent repeats with n ≥ 8 mice/group/time point. * p<0.05; ** p<0.01; *** p<0.001; **** p<0.0001

**Fig 2:**
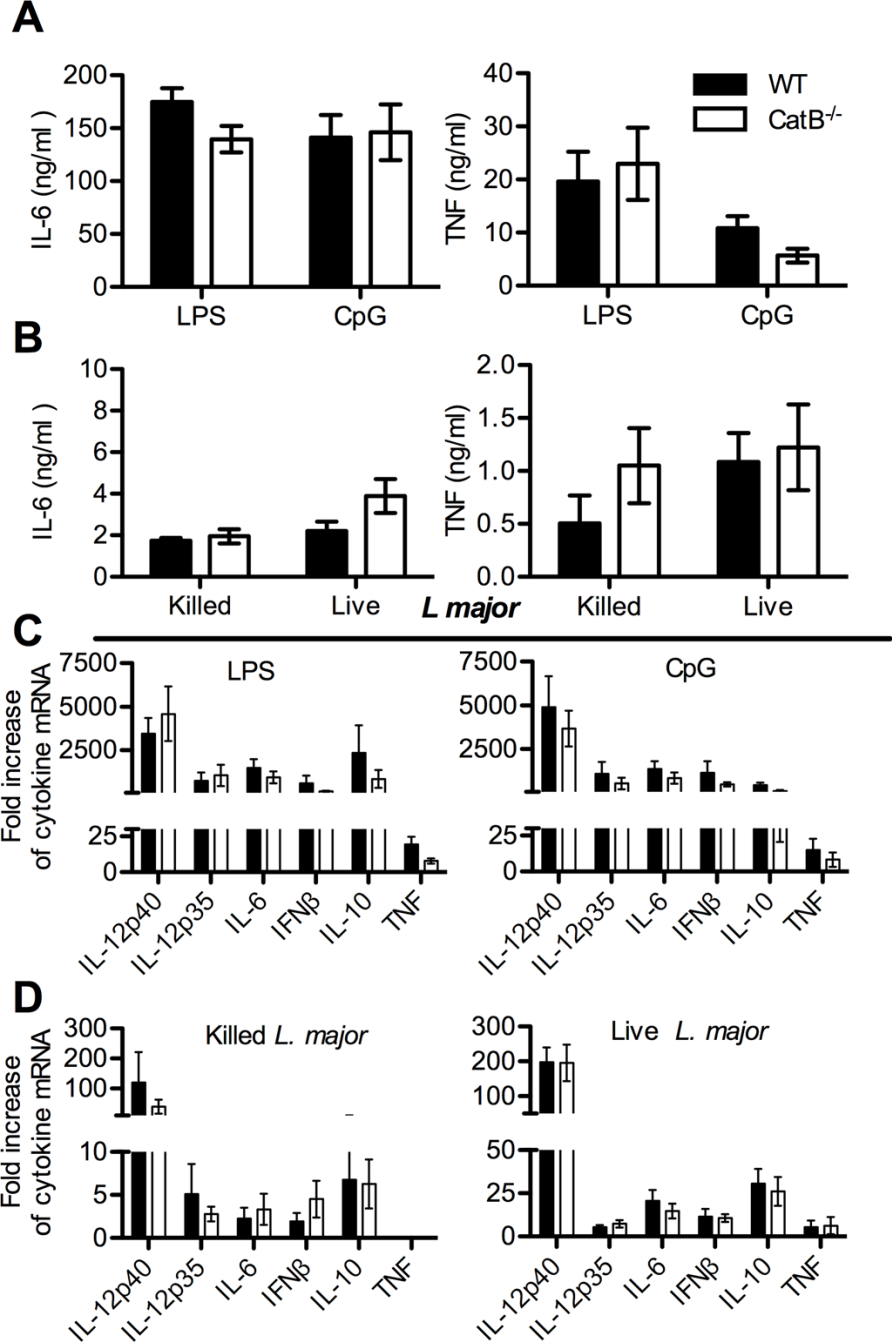
CatB^-/-^ and WT BMDCs respond similarly to TLR-agonists or *L*. *major*. BMDCs from WT and CatB^-/-^ mice were stimulated with LPS (100ng/ml) and CpG (250ng/ml) or with live or killed *L*. *major* promastigotes (1:5 MOI) for 24h to determine secreted cytokines by ELISA (**A** and **B**) or for 6h to assess mRNA by RT-PCR (**C** and **D**). **(A)** Concentration of IL-6 and TNF in supernatants of BMDCs from WT (filled bars) and CatB^-/-^ (empty bars) mice stimulated with LPS or CpG or **(B)** with live or killed *L*. *major*. **(C)** Level of expression of IL-12p40, IL-12p35, IL-6, IFNβ, IL-10 and TNF transcripts from BMDCs of WT (filled bars) and CatB^-/-^ (empty bars) mice stimulate with LPS or CpG or **(D)** with live or killed *L*. *major*. The mRNA expression levels were normalized to the expression of the HPRT gene and calculated as the n-fold difference with the level of expression in unstimulated cells. Data depicted represent the mean and SEM of three independent experiments. No statistical differences were found.

To investigate the involvement of AEP and cysteine proteases such as CatB, L and S on the development of cutaneous lesions following infection with *L*. *major*, we compared the footpad thickness between WT, AEP^-/-^, CatB^-/-^, CatL^-/-^, CatS^-/-^ and TLR9^-/-^ mice over a time-course of 49 days. Unlike TLR9^-/-^ mice, which develop significantly larger lesions and higher parasite burdens, as we previously reported [8], AEP^-/-^, and CatL^-/-^mice had similar lesion sizes and parasite burdens as compared to WT mice (Fig 1A–1C and S1 Fig). Noteworthy, CatS^-/-^ mice were slightly more susceptible to infection than WT mice (Fig 1D). These results suggest that AEP, CatL and CatS are not individually implicated in the maturation of TLR9 and do not play a major role in *L*. *major* infection. CatB^-/-^ mice, however showed a better control of infection with *L*. *major*, with footpad lesions and parasite burden in the draining lymph nodes regressing significantly faster from day 21 post-infection (p.i.) as compared to WT mice (Fig 1E and 1F). Considering the different course of infection dynamics between CatB^-/-^, WT and TLR9^-/-^, we questioned the role of this protease in the immune response to *L*.*major* infection and if it could affect TLR9 activation. Previously, we showed the importance of TLR9 for the activation of DCs by *L*.*major* DNA [8]. We used *L*. *major*, CpG and LPS to test the capacity of the BMDCs from WT and CatB^-/-^ mice to be activated by TLR9-dependent or independent stimuli. Stimulation of BMDCs from both strains of mice with CpG and LPS induced similar levels of TNF and IL-6 (Fig 2A). To assess whether cathepsin B expression is involved in the pivotal role of DCs and macrophages during *Leishmania* infection, we also stimulated these cells with heat-killed or live *L*. *major* promastigotes and found there was no significant difference in cytokine responses to *L*. *major* between cells from CatB^-/-^ and WT (Fig 2B). The same results for TNF were obtained with BMDMs from WT or CatB-/- mice (S2A Fig). Additionally, we assessed the expression of IL-12p40, IL-12p35, IL-6, IFNβ, IL-10 and TNF transcripts in BMDCs and BMDMs from WT and CatB^-/-^ mice and found no significant differences in response patterns to either *L*. *major*, CpG or LPS (Fig 2C and 2D, S2B and S2C Fig). These results suggest that the differences in control of cutaneous *L*.*major* infection observed between WT and CatB^-/-^ mice most likely do not involve DCs or macrophages and exclude a role for CatB in TLR9-dependent responses in these cells.

### Local innate immune responses in cathepsin B deficient mice reflect the faster resolution of inflammation

**Fig 3:**
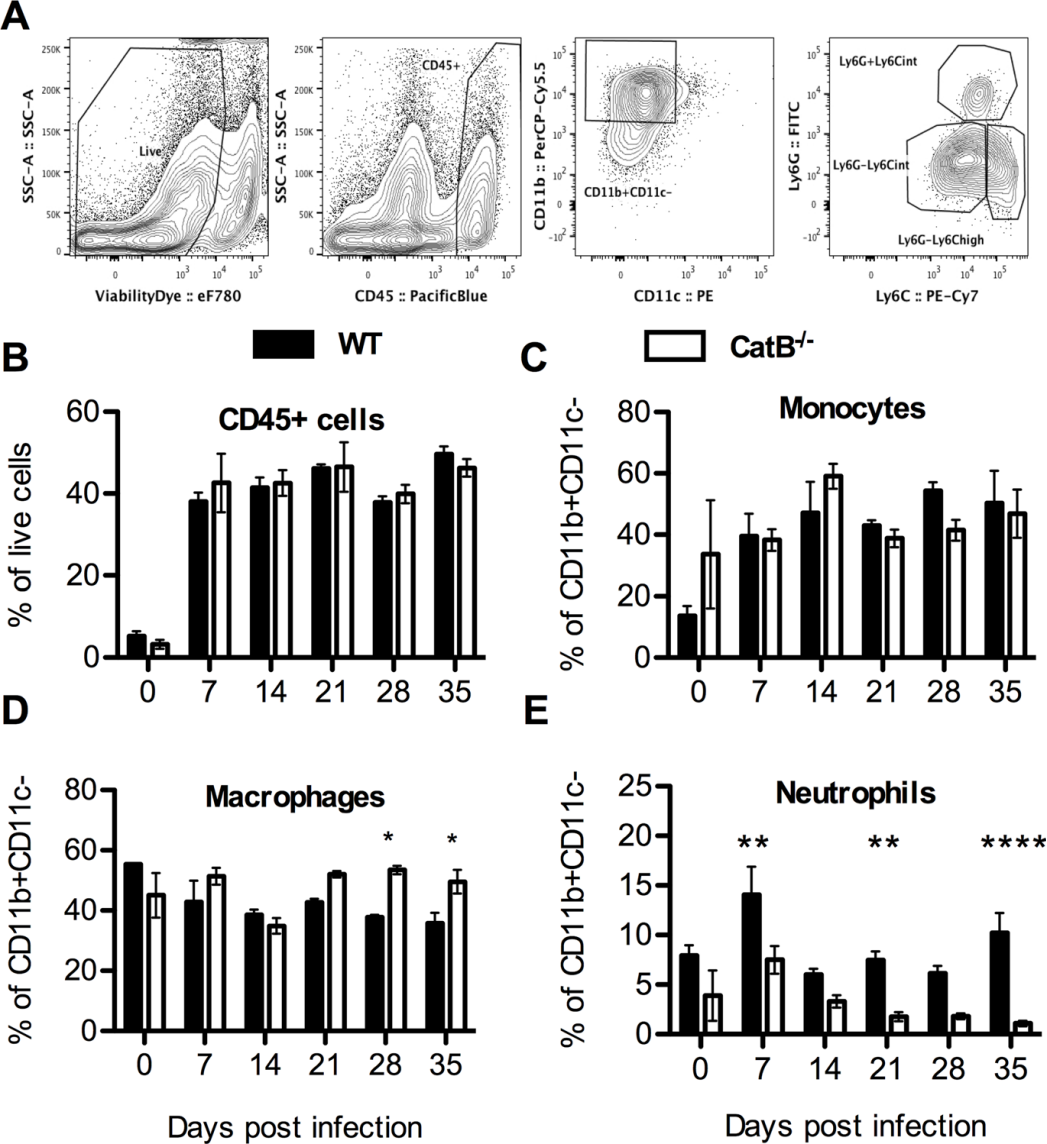
Innate immune cells in footpads of *L*. *major* infected WT and CatB^-/-^ mice. WT and CatB^-/-^ were subcutaneously inoculated with 3x10^6^ stationary phase promastigotes of *L*. *major* in the footpads and experimental read-outs were measured weekly as indicated. **(A)** Gating strategy used for FACS analysis of footpad samples prepared from *L*. *major* -infected mice. **(B)** Percentage of CD45+ cells gated on live events from footpad single cell suspensions obtained from *L*. *major* -infected WT (filled bars) and CatB^-/-^ (empty bars) mice. **(C-E)** Percentage of Ly6G^-^Ly6C^high^ monocytes **(C)**, Ly6G^-^Ly6C^int^ macrophages **(D)** and Ly6G^high^Ly6C^int^ neutrophils **(E)** gated on CD11b^+^CD11c^-^ cells from footpad single cell suspensions obtained from *L*. *major* -infected WT (filled bars) and CatB^-/-^ (empty bars) mice. Data depicted represent the mean and SEM of at least 2 independent experiments with n ≥ 4 mice/group/time point. * p<0.05; ** p<0.01; *** p<0.001; **** p<0.0001

**Fig 4:**
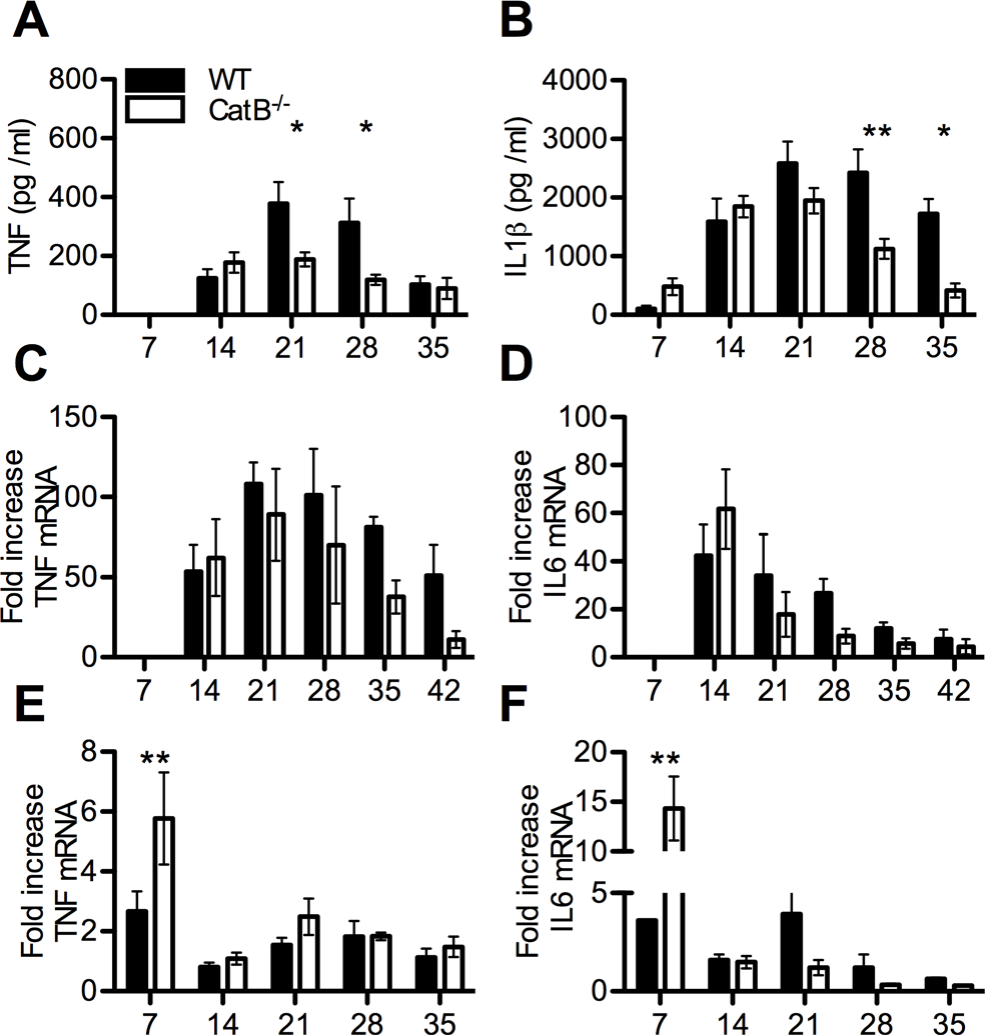
Innate immune cytokine responses of *L*. *major* infected WT and CatB^-/-^ mice. WT and CatB^-/-^ were subcutaneously inoculated with 3x10^6^ stationary phase promastigotes of *L*. *major* in the footpads and experimental read-outs were measured weekly as indicated. Levels of TNF **(A)** and IL-1β **(B)** were measured in footpads of *L*. *major* -infected WT (filled bars) and CatB^-/-^ (empty bars) mice by specific ELISA. Level of expression of TNF **(C)** and IL-6 **(D)** transcripts in footpads or TNF **(E)** and IL-6 **(F)** transcripts in lymph nodes of *L*. *major* -infected WT (filled bars) and CatB^-/-^ (empty bars) mice were measured by RT-PCR. All PCR data values are normalized to the expression of the HPRT gene. Data depicted represent the mean and SEM of at least 3 independent experiments with n ≥ 4 mice/group/time point. * p<0.05; ** p<0.01; *** p<0.001; **** p<0.0001

In order to investigate the nature of the immune response during the course of *L*. *major* infection in the two strains of mice we performed a serial analysis of multiple parameters. We first analyzed the levels of key chemokines like CXCL1 (KC), that promotes neutrophil recruitment, as well as CCL2 (MCP1) and CCL5 (RANTES), both involved in recruitment and activation of monocytes and lymphocytes, in the footpads (FPs) of infected mice. As shown in S3 Fig, ELISA analysis confirmed that the production of chemokines was higher in WT mice at days 21 and 28 p.i.. These data point to a loosening of chemokine gradients in FPs of infected CatB^-/-^ as compared to WT mice, which correlates with faster resolution. We also studied innate immune cell recruitment to the infected FPs by FACS (gating strategy depicted in Fig 3A). We found similar proportions of CD45^+^ cells in FPs of WT and CatB^-/-^ mice. Among CD45+ cells we observed similar proportions of CD11b^+^CD11c^-^Ly6C^high^ monocytes and higher frequencies of CD11b^+^CD11c^-^Ly6C^int^ macrophages in CatB^-/-^ mice, compared to WT mice, but only at late time points (Fig 3B–3D). In contrast, we found differences at the CD11b^+^CD11c^-^Ly6G^high^ neutrophil level, with significantly higher percentages of cells persisting after day 21 in WT mice (Fig 3E). We did not find differences between the percentages of macrophages, monocytes and neutrophils in the draining popliteal lymph nodes (dLNs) of WT or CatB^-/-^ mice (S4A–S4C Fig). Another innate cell population we analyzed in the dLNs were natural killer (NK) cells but again, we did not find significant differences between WT and CatB^-/-^ mice (S4D Fig). We also investigated DC content and found no significant time-stable differences in percentages of either CD11c^+^CD11b^+^ or CD11c^+^CD11b^-^ DCs in FPs or dLNs of WT and CatB^-/-^ mice (S4F–S4H Fig). Moving further, in order to decipher the local inflammatory profile governing the anti-leishmania response we determined the levels of TNF and IL-1β in the FPs. We found significantly lower levels of TNF and IL-1β in FPs of CatB^-/-^ mice at day 21 and 28 or day 28 and 35, respectively (Fig 4A and 4B). We also studied the level of expression of TNF and IL-6 mRNA in FPs and dLNs of infected mice. While we found no differences in TNF and IL-6 mRNA expression in the FPs during the whole observation period (Fig 4C and 4D), we did find significantly higher transcript levels for both cytokines in dLNs of CatB^-/-^ mice at day 7 p.i. as compared to WT (Fig 4E and 4F). Starting from day 21, inflammation significantly decreases in comparison to WT mice to promote parasite clearance and decrease of lesion size.

### Adaptive immune response mirror local inflammatory patterns and lesion dynamic

**Fig 5:**
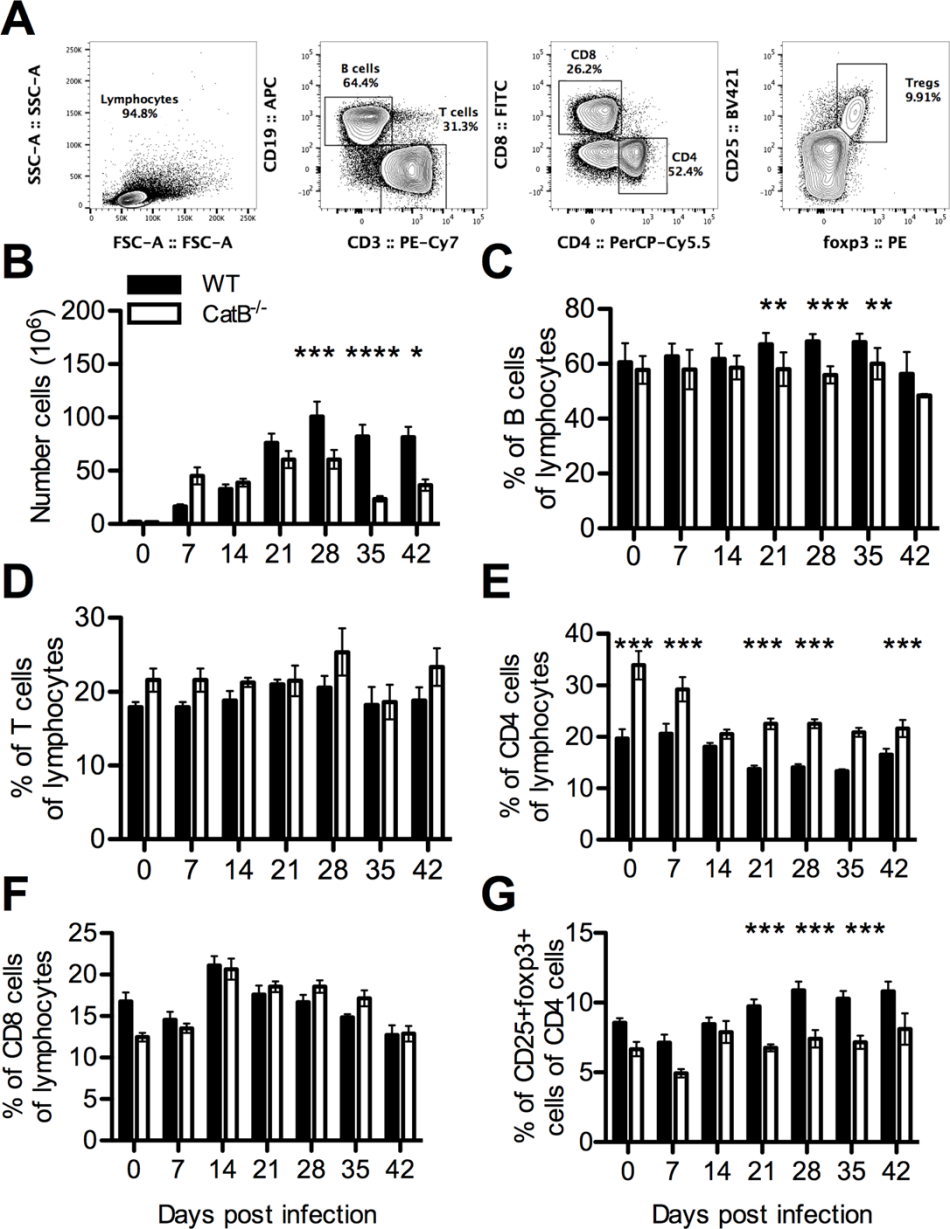
Adaptive immune cell dynamics in responses to *L*. *major* infection of WT and CatB^-/-^ mice. WT and CatB^-/-^ were subcutaneously inoculated with 3x10^6^ stationary phase promastigotes of *L*. *major* in the footpads and experimental read-outs were measured weekly as indicated. **(A)** Gating strategy used for analysis. Cell numbers in draining lymphnodes of *L*. *major* -infected WT (filled bars) and CatB^-/-^ (empty bars) mice **(B)**. Percentages of CD19+ B cells **(C)**, CD3+ T cells **(D)**, CD4+ T cells **(E)** and CD8+ T cells **(F)** gated on the lymphocyte gate and foxp3+CD25+ Tregs **(G)** gated on the CD4+ gate from lymph nodes of *L*. *major* -infected WT (filled bars) and CatB^-/-^ (empty bars) mice. Data depicted represent the mean and SEM of at least 3 independent experiments with n ≥ 4 mice/group/time point. * p<0.05; ** p<0.01; *** p<0.001; **** p<0.0001

**Fig 6:**
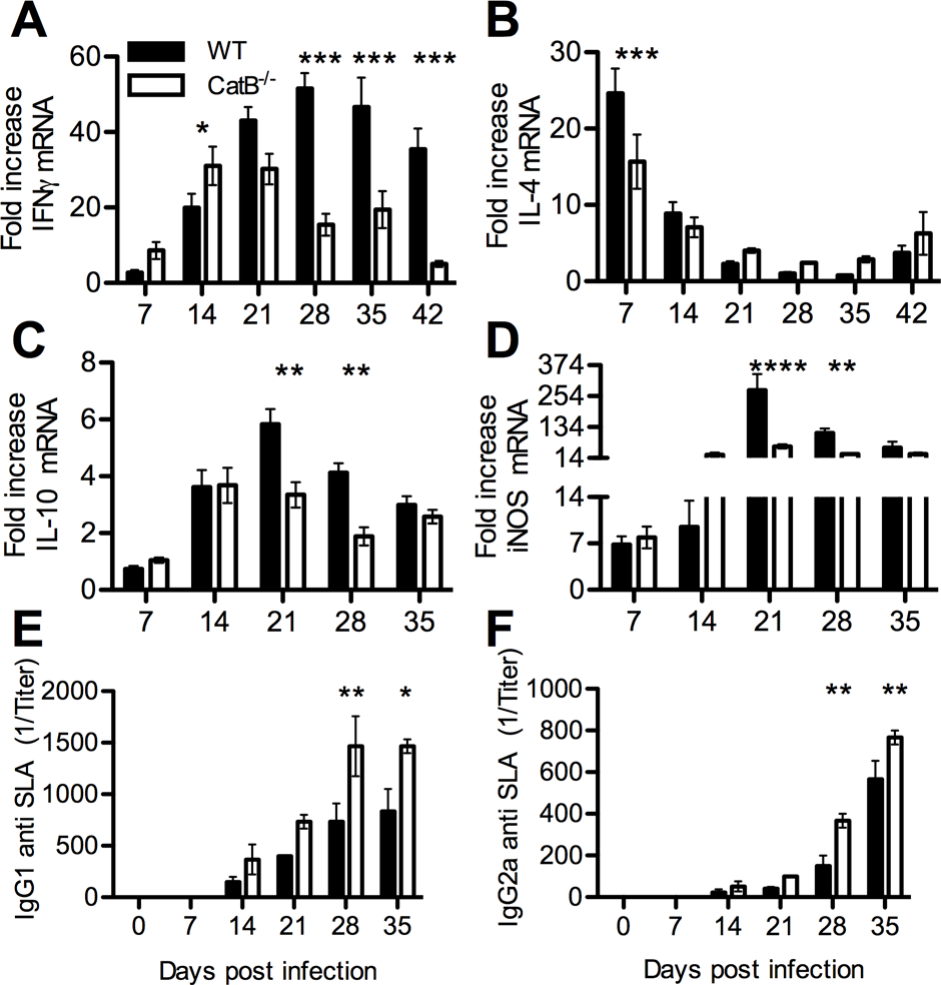
Protective local cytokine responses develop faster and resolve sooner in CatB^-/-^ mice. WT and CatB^-/-^ were subcutaneously inoculated with 3x10^6^ stationary phase promastigotes of *L*. *major* in the footpads and experimental read-outs were measured weekly as indicated. Expression levels of IFNγ **(A)**, IL-4 **(B)**, IL-10 **(C)** and iNOS **(D)** transcripts were determined in lymph nodes of *L*. *major* -infected WT (filled bars) and CatB^-/-^ (empty bars) mice by RT-PCR. Values are normalized to the expression of the HPRT gene. Levels of SLA-specific IgG1 **(E)** and IgG2a **(F)** were determined by ELISA on serum samples harvested from *L*. *major* -infected WT (filled bars) and CatB^-/-^ (empty bars) mice. Data depicted represent the mean and SEM of at least 3 independent experiments with n ≥ 4 mice/group/time point. * p<0.05; ** p<0.01; *** p<0.001; **** p<0.0001

Because proliferation of lymphocytes in dLNs remains an absolute requirement for adaptive immunity in cutaneous leishmaniasis, we studied the dynamics of T and B lymphocytes in WT and CatB^-/-^ mice during the course of infection (gating strategy in Fig 5A). Cell numbers in dLNs, the majority of which are lymphocytic, increase dramatically during the course of infection. Especially in WT mice, dLN cell numbers were 50 to 100 times higher compared to uninfected mice. These numbers peaked at day 28 in WT mice and slowly decreased thereafter (Fig 5B). In contrast, cellularity of dLNs in CatB^-/-^ mice peaked at day 21 and decreased at a faster rate compared to WT mice. Phenotypically, we observed a constantly lower percentage of CD19^+^ B cells in the dLNs of CatB^-/-^ mice reaching significance from day 21 (Fig 5C). While CD3^+^ T cell percentages were not different between the two strains of mice, CatB-/- mice had higher CD4^+^ T cell percentages compared to WT (Fig 5D–5F). Among CD4^+^ T cells, we observed significant lower foxp3^+^CD25^+^ regulatory T cells (Tregs) in CatB^-/-^ from day 21 onward (Fig 5G). To complete our investigation of adaptive responses during *L*. *major* infection, we explored the dynamics of T cell related cytokines in dLNs and FPs of infected mice. We compared the expression profile of Th1 (IFNγ) and Th2-associated (IL-4 and IL-10) mRNAs in dLNs of WT and CatB^-/-^ mice during infection since we found no detectable transcripts in the FPs. While we observe increased IFNγ expression at early moments in CatB^-/-^ mice, this trend is quickly lost past day 21 p.i. reaching significantly lower levels compared to WT from day 28 p.i. onward (Fig 6A). Interestingly, this tendency of CatB^-/-^ to express higher levels of IFNγ like at day 14 p.i. was corroborated by a significantly lower expression of IL-4 transcripts in these mice at day 7 p.i. (Fig 6B). IL-4 expression further decreased and showed no significant differences during the rest of the observation period. We also observed a lower IL-10 transcript expression in CatB^-/-^ mice around day 21–28 p.i. (Fig 6C) Since the IFNγ-inducible Nitric Oxide Synthase (iNOS) axis is well recognized as an effector mechanism in leishmaniasis we also monitored the levels of iNOS transcripts in dLNs and FPs of infected mice. CatB^-/-^ mice had significantly lower levels of iNOS in the dLNs compared to WT from day 21 to 28 p.i. and a similar tendency further on (Fig 6D). Interestingly, iNOS transcripts in the FPs were highly upregulated throughout the infection (S5A Fig), with differences between mouse strains showing a similar trend as like in dLNs but without reaching significance.

Additionally, we performed a serial analysis of soluble *Leishmania* antigen (SLA)-specific immunoglobulins and observed an increased level of total IgGs, as well as increased levels of IgG1 and IgG2a in sera of CatB^-/-^ mice as compared to WT, with differences reaching statistical significance at days 28 and 35 (Fig 6E and 6F). The IgG2a/IgG1 ratio however showed no significant differences between WT and CatB^-/-^ mice throughout the observation period (S5B Fig). Altogether these data point to a faster and more efficient induction of a protective Th1 response in CatB^-/-^ as compared to WT accompanied by a faster transit to a resolution phase which correlates with a lower presence of Tregs and IL-10.

### Similar control of Th1 responses by WT and CatB^-/-^ DCs

**Fig 7:**
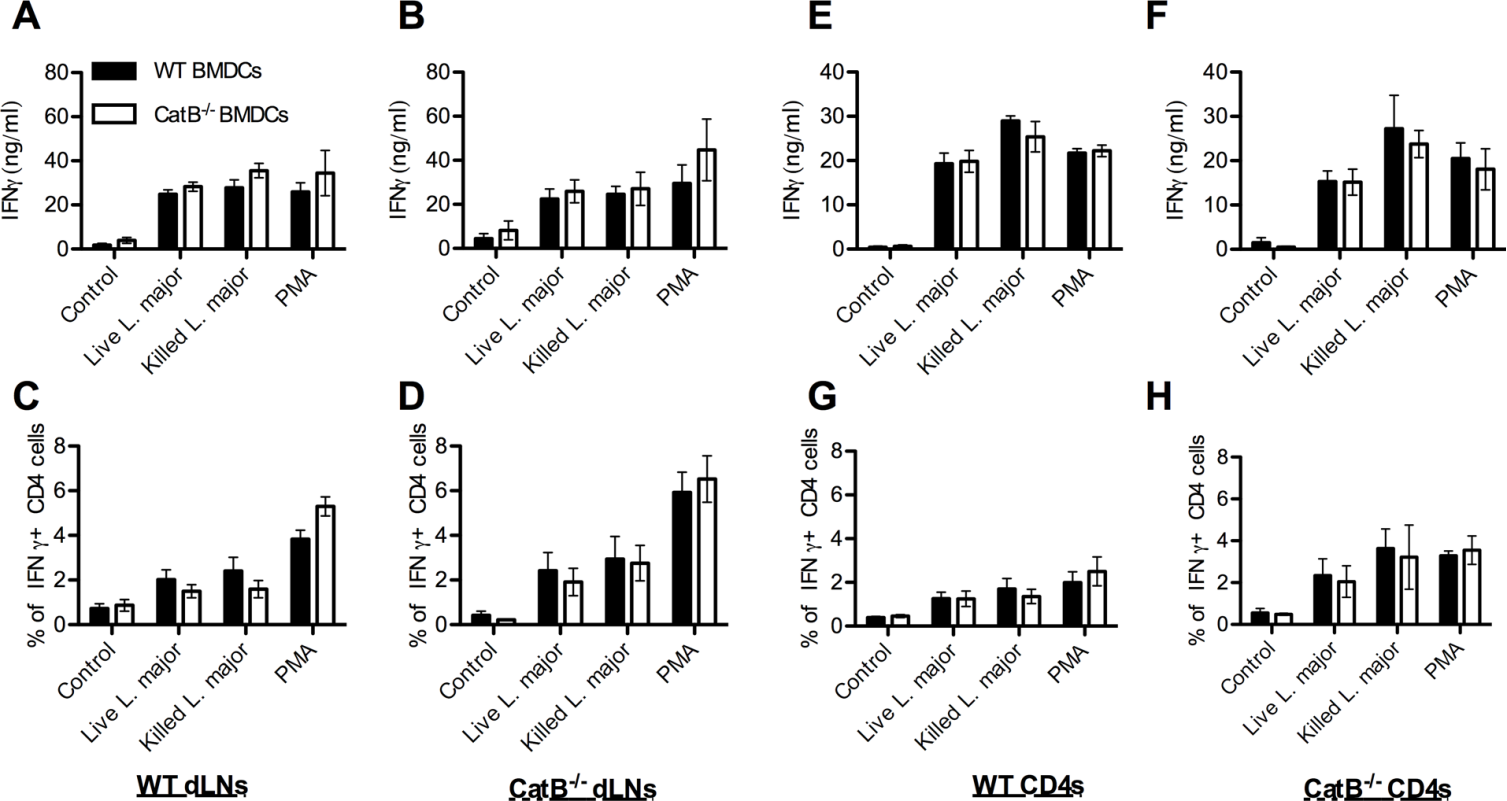
Similar antigen presentation capacity of BMDCs from WT and CatB^-/-^ mice. WT and CatB^-/-^ were infected subcutaneously with 3x10^6^ stationary phase promastigotes of *L*. *major* in the footpads. On days 21 and 28, lymph nodes were harvested and whole cells or purified CD4 cells from these were co-cultured overnight with WT or CatB^-/-^ BMDCs that were non-stimulated as control or activated by PMA, killed or live *L*. *major*. Supernatants were harvested for cytokine determination by specific ELISA and unadherent cells were harvested for intracellular cytokine staining by FACS. **(A-B)** IFNγ levels secreted by lymph node (dLNs) cells and (**C-D)** percentage of IFNγ+ cells gated on CD4+ cells in these dLNs from *L*. *major*-infected WT **(A, C)** or CatB^-/-^ **(B, D)** mice after co-culture with WT (filled bars) or CatB^-/-^ (empty bars) BMDCs. **(E-F)** IFNγ levels secreted by CD4+ cells and **(G-H)** percentage of IFNγ+ cells gated on CD4+ cells from purified CD4+ cells of *L*. *major*-infected WT **(E, G)** or CatB^-/-^ **(F, H)** mice that were co-cultured with WT (filled bars) or CatB^-/-^ (empty bars) BMDCs. Data depicted represent the mean and SEM of at least 3 independent experiments with n ≥ 4 donor mice/group.

While previous studies hypothesized that inhibition of CatB during *L*. *major* infection would lead to changes in Th cell polarization due to difference in antigen presentation [18,19], we sought to investigate whether *L*.*major* activated WT and CatB^-/-^ DCs had the same ability to stimulate antigen-specific T cells from *L*. *majo*r infected mice. To this aim, we took dLNs from day 21 and 28 infected mice and we co-cultured these cells with *L*. *major* antigen-loaded BMDCs to determine IFNγ production by specific ELISA and intracellular staining. As shown in Fig 7A–7D, we observed similar IFNγ production regardless of the origin of the BMDCs used for stimulation. To restrict antigen presentation to class II MHC, we performed similar experiments using purified CD4^+^ T cells from dLNs at days 21 and 28 p.i. and again observed no BMDC-dependent differences between antigen-presentation capacity of WT or CatB^-/-^ mice (Fig 7E–7H). We observed no detectable IFNγ secretion under control conditions with un-stimulated BMDCs or naïve CD4^+^ T cells. These data indicate that DCs from WT and CatB^-/-^ mice are equally capable of presenting *L*. *major* antigens in a MHC II context, suggesting that antigen presentation does not likely contribute to the differences seen in infection clearance between the two strains.

### CatB^-/-^ T cells confer protective phenotype upon adoptive transfer into lymphopenic mice

**Fig 8:**
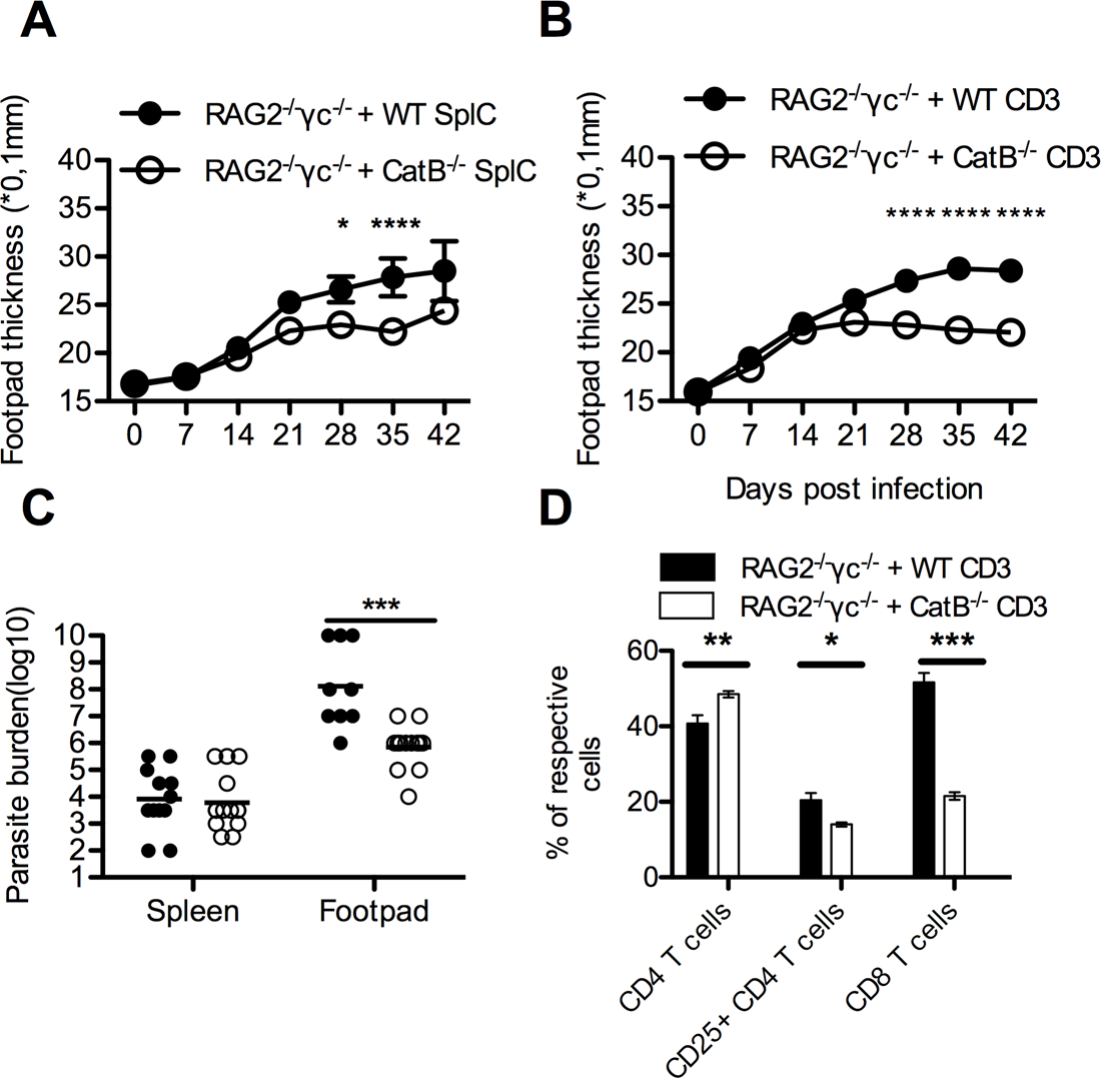
CatB^-/-^ T cells mediated protective phenotype upon adoptive transfer into alymphpoid RAG2^-/-^γc^-/-^ mice. Splenocytes or purified CD3+ T cells naïve WT and CatB^-/-^ were transferred i.v. (5x10^6^ cells/100ul for splenocytes or 10^6^ cells/100ul for purified CD3+ cells) into RAG2^-/-^γc^-/-^ mice, seven days before infection. Recipient mice were infected subcutaneously with 6x10^6^ stationary phase promastigotes of *L*. *major* in the footpads and experimental read-outs were assessed weekly as indicated. (**A)** Mean footpad thickness (±SEM) of infected RAG2^-/-^γc^-/-^ mice reconstituted with splenocytes of donor mice (WT–filled circle; CatB^-/-^- empty circle). (**B)** Mean footpad thickness (±SEM) of infected RAG2^-/-^γc^-/-^ mice reconstituted with purified CD3 T cells of donor mice (WT–filled circle; CatB^-/-^- empty circle). **(C)** Logarithm of limit dilution for parasite burden from footpads and spleens of RAG2^-/-^γc^-/-^ mice reconstituted with purified CD3 T cells of donor mice (WT–filled circle; CatB^-/-^- empty circle). **(D)** Percentage of respective cell populations identified in spleens of RAG2^-/-^γc^-/-^ mice reconstituted with purified CD3 T cells of donor mice (WT–filled circle; CatB^-/-^- empty circle). Respective populations are gated on CD45+ cells out of live events. Figures depict representative data pooled from 3 independent repeats with n ≥ 5 mice/group * p<0.05; ** p<0.01; *** p<0.001; **** p<0.0001

Data presented so far do not support a role for APCs in the observed differences between CatB^-/-^ and WT mice during *L*. *major* infection but rather one for lymphocytes, given the more efficient induction of a protective Th1 response and rapid decline of inflammatory cells in CatB^-/-^ dLNs. To address this issue specifically we reconstituted RAG2^-/-^γc^-/-^ mice with splenocytes (SplC) from either WT or CatB^-/-^ mice (RAG2^-/-^γc^-/-^ + WT or RAG2^-/-^γc^-/-^ + CatB^-/-^ SplC, respectively) and measured FP thickness after infection with *L*. *major*. RAG2^-/-^γc^-/-^ + CatB^-/-^ SplC showed less FP swelling compared with mice reconstituted with WT cells at days 21–35 p.i. (Fig 8A). These data argued for predominant role of lymphocytes in the immune advantage of CatB^-/-^ mice during *L*. *major* infection, but could not pinpoint a specific cell population in cause. We thus went further and repeated the reconstitution experiments with purified CD3+ T cells (RAG2^-/-^γc^-/-^ + WT or RAG2^-/-^γc^-/-^ + CatB^-/-^ CD3s, respectively). Similar to the splenocyte transfer experiments, we found that RAG2^-/-^γc^-/-^ + CatB^-/-^ CD3s had milder FP swelling compared to RAG2^-/-^γc^-/-^ + WT CD3s arguing for a T cell-related difference (Fig 8B). Since RAG2^-/-^γc^-/-^mice do not have dLNs and leishmania parasites disseminate systemically in these mice [32], we assessed cellularity and parasite burdens in the spleens. At day 42 p.i. the number of viable parasites in spleens from RAG2^-/-^γc^-/-^ + CatB^-/-^ CD3s was 1–2 orders of magnitude lower than in RAG2^-/-^γc^-/-^ + WT CD3s, whereas in the FP the differences were more than 3–4 orders of magnitude less in the same direction reaching statistical significance (Fig 8C). The cellular composition of the spleens in reconstituted RAG2^-/-^γc^-/-^ mice reflected the origin of the transferred cells, but also mirrored the situation found in WT or CatB^-/-^ mice post infection, with more CD4 and less CD25^+^CD4^+^ cells (Fig 8D). Interestingly, the spleens of naïve mice showed that CD8 cells are underrepresented in CatB^-/-^ mice (S6 Fig), and we observed a significantly lower percentage of CD8 T cells in RAG2^-/-^γc^-/-^ + CatB^-/-^ CD3s.

**Fig 9:**
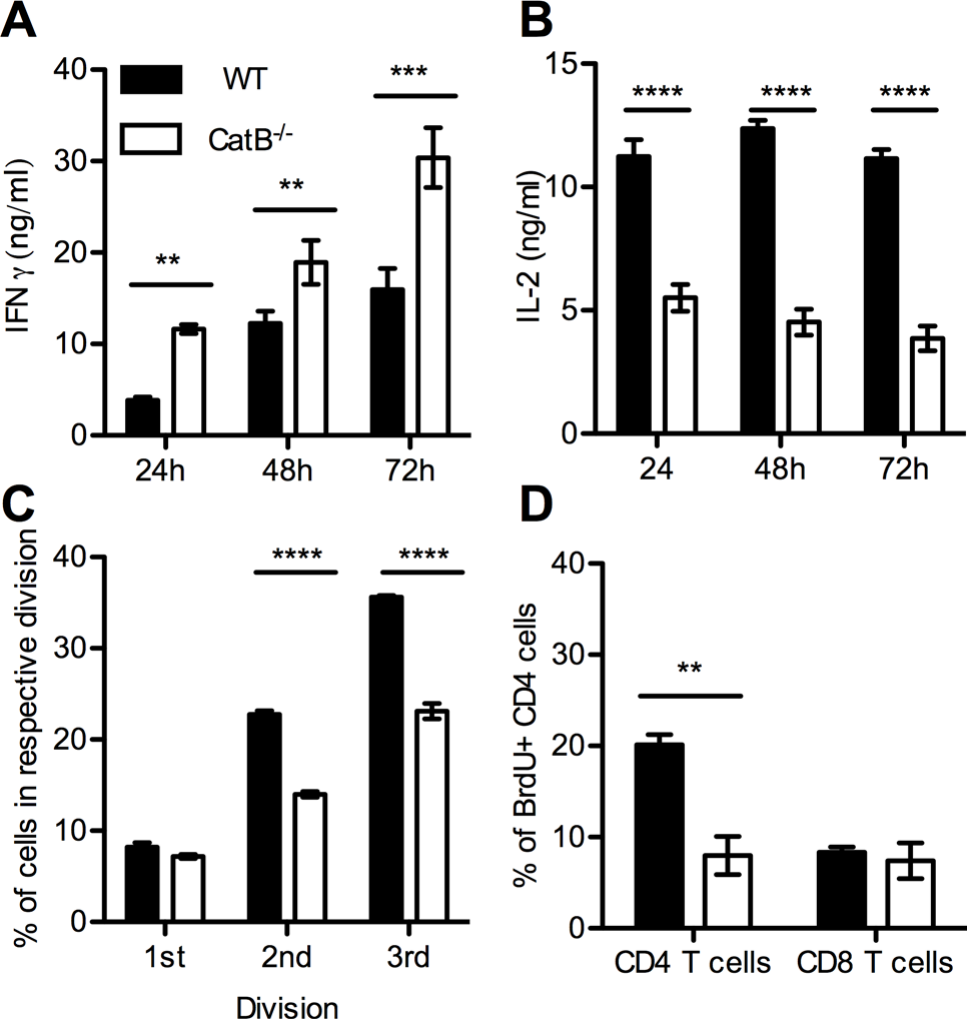
CatB^-/-^ T cells secrete more IFNγ but less IL-2 and have impaired proliferation as compared to WT T cells. *In vitro* CD3 T cells purified from spleens of naïve WT and CatB^-/-^ mice were labelled with CFSE or not and stimulated overnight with PMA or with anti-CD3. Supernatants were harvested for cytokine detection at 24, 48 and 72h and CFSE dilution was assessed by FACS at 72h. Concentration of IFNγ **(A)** and IL-2 **(B)** in supernatants of PMA stimulated CD3 T cells from WT (filled bars) and CatB^-/-^ (empty bars) mice. **(C)** Percentage of CD4 cells that have undergone 1, 2 or 3 divisions based on CFSE labelling at 72h after stimulation with plate-bound anti-CD3 of purified T cells from WT (filled bars) and CatB^-/-^ (empty bars) mice. *In vivo* purified CD3 T cells were transferred into recipient RAG2^-/-^γc^-/-^ mice and BrdU assay was performed on day 21 after transfer. **(D)** Percentage of BrdU+ CD4 and CD8 cells identified among CD45+ cells in spleens of RAG2^-/-^γc^-/-^ mice reconstituted with purified CD3 T cells from WT (filled bars) and CatB^-/-^ (empty bars) mice at day 21 after transfer. Figures depict representative data pooled from 3 independent repeats with n ≥ 5 mice/group * p<0.05; ** p<0.01; *** p<0.001; **** p<0.0001

Additionally in order to identify the T cell-intrinsic differences that could account for the observed phenotypes, we assessed cytokine-production and proliferation capacity of isolated T cells *in vitro*. Following stimulation with PMA, CatB^-/-^ T cells produced significantly more IFNγ than WT T cells but significantly less IL-2 (Fig 9A and 9B). To assess proliferative capacity, we stimulated CFSE-labeled purified T cells with anti-CD3 for 72 hours and analyzed CFSE dilutions by FACS. As depicted in Fig 9C, CatB^-/-^ CD4 cells have a lower proliferative capacity at 72h as compared to WT T cells. Moreover, we assessed the *in vivo* proliferation capacity purified T cells by measuring BrdU incorporation at 21 days after transfer into RAG2^-/-^γc^-/-^ mice. We found significantly higher incorporation of BrdU in WT CD4 cells as compared to CatB^-/-^, but no differences at the CD8 level (Fig 9D).

In summary, our data sustain a role for cathepsin B at the T cell level, allowing a higher proliferation rate but a lower IFNγ secretion capacity. In the case of *L*. *major* infection, this might make WT mice develop a later but more sustained Th1 type response, maintaining inflammatory lesions for a longer time.

## Discussion

We previously showed that TLR9 plays a critical role in resistance to *L*. *major* that is dependent on a parasite DNA–TLR9 –DC–Th1 axis [8,29]. The work presented here aimed at investigating the functional consequences that different proteases, reported to intervene in the functional maturation of TLR9, might have on the evolution of cutaneous *L*. *major* infection. Using mice genetically deficient for AEP, CatB, CatL and CatS we found that only CatB^-/-^ were significantly different from WT mice. These mice showed a faster dynamic and increased efficiency of cutaneous lesion resolution and clearance of *L*. *major* parasites. Previous studies on the role of cathepsins during *L*. *major* infection revealed different effects of cathepsin inhibition. Targeting cathepsin B by specific inhibitors was reported to be protective in BALB/c mice but with no effect on DBA/2 mice [18]. In agreement with these studies we report a protective phenotype in CatB^-/-^ mice. CatL inhibitors on the other hand, were reported to exacerbate disease in susceptible BALB/c and resistant DBA/2 mice [17,33]. We find no effect of CatL, although factors like specificity of the inhibitors as opposed to genetic deletion might be responsible for the different results. Our results complete and expand on existing literature by the use of AEP and CatS knockout mice, where we find no major difference in disease progression and parasite clearance. To our knowledge this is the first report on the role of these proteases during *L*. *major* infection. The slightly higher susceptibility, which we observed in the CatS^-/-^ mice, might be linked to an incomplete maturation of TLR9 or a breakdown in cleavage of the MHCII invariant chain, as previously reported for this cathepsin [14,34]. Since none of the AEP^-/-^, CatL^-/-^, CatS^-/-^ or CatB^-/-^ mice reproduced the susceptibility of TLR9^-/-^ to *L*. *major* infection in terms of lesion size and parasite clearance we concluded that these proteases are not individually involved in TLR9 functional maturation. However, we can also not exclude a compensatory role for any given cathepsin in the absence of the other.

Looking for a role of cathepsin B in the response to TLR9 stimulation, we assessed APC cytokine responses of WT and CatB^-/-^ mice to CpG and *L*. *major*. We found no influence of CatB on responsiveness of BMDCs or BMDMs to TLR9-dependent or independent stimulation in terms of cytokine transcription or production. In opposition to our finding, a recent study reported that CatB deficiency affects both antigen presentation and IL-12 production from APCs upon stimulation with *L*. *major* [19]. While we did not assess protein levels of IL-12, we did not observe significant differences between WT and CatB^-/-^ DCs in terms of the transcriptionally regulated expression of IL-12p35 [35] to live or killed *L*. *major* or other stimulants like LPS and CpG. However minor variations in experimental conditions like composition of culture media, type of serum and growth factors used might also be responsible of the observed differences. We also find similarities with the aforementioned work, like no difference in IL-6 production in response to *L*. *major*. Differential harvest times might also explain why we see no differences in TNF response to LPS, as reported by Ha *et al* [36], however as the authors themselves mention, TNF can probably be secreted through a cathepsin B independent pathway. Interestingly, membrane-associated TNF was shown to be sufficient to protect mice during leishmania infection [37]. But a subsequent study showed that this was also dependent on the *L*. *major* strain used [38]. Taking this into account, the differences we find in relation to earlier work on the influence of CatB on the APC response to *L*. *major* [19], might also be due to the different strains used (LV39 vs MHOM/IL/81/FE/BNI). All in all, our results suggest that in our model, dendritic cell or macrophages are not likely to be the source of the differences observed in the control of *L*. *major*.

Cysteine protease cathepsin L and B function as antigen processing proteases and modulate the processing pattern of *L*. *major* antigen (SLA). It was shown that the digestion of SLA by a mitochondria /lysosome fraction was slightly influenced by the presence of an inhibitor for catepsine B or L but overall, the degradation profile of the antigen remains similar [17,18,33,39]. Indeed, degradation was very partial and the size of the fragments still very far from those of the peptides which are presented. To address this question, we assessed the antigen presenting capacity of BMDCs from WT and CatB^-/-^ mice. We found no difference between the antigen presenting capacity of cells from both mice strains; BMDCs being as efficient at triggering IFNγ secretion from *in vivo* primed T cells. As originally described, we found that cathepsin B is dispensable for MHC class II presentation [25]. Once again we saw no indication of a role of APCs (DCs) in the difference in anti-leishmanial T cell responses, although we cannot exclude a difference in antigen presentation by other cells like B cells [40].

The resistant C57BL/6 genetic background of WT and CatB^-/-^ mice to *L*. *major* infection allowed us to compare immune parameters over a long course of infection in a high-dose and relevant model of self-resolving leishmaniasis. We defined the soluble mediators and cellular components of the inflammatory response. Overall, WT mice responded to *L*. *major* with a delayed, enhanced and more sustained inflammation as compared to CatB^-/-^. This inflammatory pattern was characterized by higher local chemokine levels accompanied by a significantly increased infiltration with neutrophils in both FPs and dLNs that persists beyond day 21 of infection in WT mice. High neutrophil numbers at the site of inoculation reflect the inflammatory state but have also been shown to correlate with immune escape in leishmaniasis [41], neutrophils being considered as a Trojan horse used by the parasite to evade the immune system [42]. The higher levels of IL-6 and TNF we find in dLNs of CatB^-/-^ at day 7 p.i. are interesting, yet difficult to correlate with a response that might favour Th1 priming. Although the role of IL-6 during *L*. *major* infection has been previously investigated, it was shown to have no major effect or at least not on early responses [43–45] TNF however, was shown to have a clear protective role in L. major infection, with higher TNF levels in C57BL/6 mice compared to BALB/c [37,46]. We did not observe impairment in IL-1β secretion in CatB^-/-^ mice, these results being consistent with a cathepsin B-independent activation of IL-1β, especially since multiple cathepsins have been implicated in NLRP3-mediated IL-1β activation [47]. Our results thus point to a minor influence of CatB deficiency on the innate response during *L*. *major* infection.

A delicate balance between Th1 and Th2 is supported as the turning point for anti-leishmanial immunity and this conclusion has benefited greatly by studies in prototypical Th1 and Th2 mice, C57BL/6 and BALB/c, respectively [48,49]. An early Th2 has been reported to develop in C57BL/6 mice, which is afterwards shut down and dominated by a protective Th1 [50]. Accompanying the favorable phenotype of CatB^-/-^ mice, we observed that an adaptive Th1 response started at earlier time-points and was resolved faster as compared to WT mice. Previous reports on use of cathepsin B inhibitors have shown that *in vivo* treatment of infected BALB/c mice can suppress the development of a deleterious Th2 [18]. Consistent with these early studies, we observed a tendency of CatB^-/-^ mice to express higher levels of IFNγ and lower levels of IL-4 in the early phases of disease. This trend was lost after day 21 when CatB^-/-^ Th1 markers fall below those of WT mice. We were not able to detect transcript for these cytokines in the FPs, probably because of the low numbers of T cell present at this site as compared to the dLNs. We did however find very high iNOS transcript upregulation in the FPs. While WT mice maintain higher levels of Th1 cytokines and increased cellularity in the dLNs, they also maintain a relatively high size of FP lesions and parasite burdens. These observations have already been described and attributed to anti-inflammatory mechanisms like Treg expansion and IL-10 secretion which allow persistence of the parasite [51]. We observed a difference in the representation of T cell populations in CatB^-/-^ mice, characterized by a higher percentage of CD4, among which a lower percentage of cells were CD25+foxp3+ Tregs. This difference was present in naïve mice and maintained during the course of infection indicating that the equilibrium between effector and regulatory T cells as described by Belkaid *et al*. [52] is higher in C57BL/6 mice than in CatB^-/-^, which might explain the faster development and regression of the inflammatory response in these mice. To further evaluate the immune status of infected mice, we examined the proportion of *L*. *major-*specific IgG subclasses. Except for a higher abundance of antibodies in CatB^-/-^ mice, we found no indication of a preferential skewing of the IgG2a/IgG1 ratio. Since we did not observe higher, but in fact lower percentages of B cells in CatB^-/-^ mice during later stages of the disease, we believe that a qualitative difference in Th-responses at early moments might induce the differences we observe in IgG levels.

Cathepsin B is one of the most abundant cysteine proteases not only present in lysosomes of APCs but also in B and T lymphocytes [21,22,53–55], and has been described to be implicated in different inflammatory disease [56,57]. Lysosomal proteases were believed to be mainly involved in different types of cell death. Aside from the role of cathepsin B in promoting cell death, a different line of research suggested that surface cathepsine B protects cytotoxic lymphocytes and NK cells from self-destruction after degranulation [22]. These results were obtained with highly specific cathepsin B inhibitors, but were not confirmed by Baran *et al* [23], using CatB^-/-^ mice. In addition it has been shown that *in vitro* supra optimal activation induces apoptosis of T cells due to release of catalytically active CatB and CatL in the cytosol [53].

Considering the possible effects of cathepsin B on lymphocytes, we turned to a system in which we could prove the involvement of lymphocytic cells in the CatB phenotype. We initially reconstituted RAG2^-/-^γc^-/-^ mice with splenocytes from either WT or CatB^-/-^ and observed FP thickness after infection with *L*. *major*. RAG2^-/-^γc^-/-^ mice reconstituted with CatB^-/-^ splenocytes developed significantly smaller lesions throughout the course of infection compared with mice reconstituted with WT cells. While this result argues for a mostly lymphocytic origin of the observed differences, we could not exclude the influence of small myeloid populations in the spleen. Further experiments led to similar observations with the use of isolated CD3+ T cells. These results argue that, at least in our RAG2^-/-^γc^-/-^ reconstitution system, B cells and NK cells along with other non-T cell populations in the spleen, do not contribute much to the development of local inflammation during *L*. *major* infection. We found significantly lower parasite burdens in the FP of RAG2^-/-^γc^-/-^ mice reconstituted with CatB^-/-^ T cells as compared to WT T cells. As previously described [32], RAG2^-/-^γc^-/-^ mice fail to contain systemic dissemination of leishmanial parasites due to lack of lymph nodes, which might also account for the lack of resolution we observed in these mice. Differences in cellular phenotype similar to those found between dLNs of WT and CatB^-/-^ mice were also found in the spleens of reconstituted mice, as a higher percentage of CD4 + cells with significantly fewer CD4+CD25+ cells among them. In RAG2^-/-^γc^-/-^ mice reconstituted with CatB^-/-^ T cells we observed a lower percentage of CD8 + as compared to WT in spleen, which was in accordance to the original phenotype of the transferred cells. The lower level of CD8 could explain the better resolution of the inflammatory response as previously suggested by Belkaid *et al*, which showed that CD8 T cells contribute to inflammation in a RAG2^-/-^ reconstitution model [58].

These points led us to study the cytokine production capacity and the ability to proliferate of T cells. We found that CatB^-/-^ T cells have an intrinsic capacity to secrete more IFNγ but are less able to produce IL-2 and proliferate *in vitro*. Altogether, the data underline that intrinsic differences in the T cell compartment exist between WT and CatB^-/-^ mice which evoke a precocious Th1 response, probably as a result of a higher IFNγ production capacity, and faster resolution of inflammation which could be attributed to the lower proliferative capacity of CatB^-/-^ CD3 T cells. We cannot at this moment say if the differences in T cell reactivity imply a cell-intrinsic role for CatB or are the result of development and selection of T cells in a CatB^-/-^ -environment. Given the ubiquitous expression of CatB and the many functions attributed to this protease, identifying a precise mechanism of action might prove very difficult. In addition to the differences in cytokine secretion capacity and proliferation we report for T cells, cathepsin B might affect other processes that may influence *L*. *major* disease progression like the reported role in cleavage of chemokines or IL-1 processing [47,59].

Unanswered questions remain on the source of these functional differences within the T cell population. The imbalance in CD8, CD4 and Treg might be a first reason to consider, followed by the individual competence of each of these populations. The increased inflammatory capacity correlates with a possible defect in Treg suppressive capacity.

Cathepsin B has already been proposed as a therapeutic target for inflammatory diseases, and for leishmaniasis, and our work brings forth new evidence on the effects that cathepsin B inhibition might have at the T cell level, and sets the stage for future investigation.

## Supporting Information

S1 Fig Parasite burdens of WT, CatL-, CatS- and AEP-knockout mice. WT, CatS^-/-^, CatL^-/-^ and AEP^-/-^ mice were subcutaneously inoculated with 3x10^6^ stationary phase promastigotes of *L*. *major* in the footpads and experimental read-outs were measured at indicated time-points. Logarithm base 10 of limit dilution for parasite burden from footpads of infected mice. Data are presented as mean+SD. n = 3–7 mice per group/time-point.
(PDF)

S2 Fig CatB^-/-^ and WT BMDMs respond similarly to LPS, CpG, killed or live *L*. *major*. BMDMs from WT and CatB-/- mice were stimulated with LPS (100ng/ml), CpG (250ng /ml), live or killed L. major promastigotes (1:5 MOI) to determine TNFα production by ELISA (**A**) and to assess cytokine mRNAs expression by RT PCR (**B** and **C**).
(PDF)

S3 Fig Chemokines responses of L. major infected WT and CatB^-/-^ mice. WT and CatB^-/-^ were inoculated subcutaneously in the footpads with 3x10^6^ stationary phase promastigotes of *L*. *major*. Footpads were harvested at day 21 and 28 post infection. Levels of CXCL1 (A), CCL2 (B) and CCL5 (C) were measured in the supernatants of footpads by ELISA.
(PDF)

S4 Fig Innate immune cells in lymphnodes (dLNs) or footpads (FPs) of L. major infected WT and CatB^-/-^ mice. Mice were harvested weekly as indicated. (**A-C**). Lymphnode samples prepared from *L*. *major*-infected WT (filled bars) and CatB^-/-^ (empty bars) mice. CD11b^+^ CD11c^-^ were gated on live events from lymphnodes single cell suspensions and FACS analysis of the percentage of (**A**) Ly6G^-^Ly6C^high^ monocytes (**B**) Ly6G^-^Ly6C^int^ macrophages and (**C**) Ly6G^high^Ly6C^int^ neutrophils were performed, (**D**) NK were gated on lymphocytes. (**E-F**) In footpads (FPs) of L. major infected WT and CatB^-/-^, CD45^+^ were gated on live events for facs analysis of DCs (**E**) CD11b^+^CD11c^+^ and (**F**) CD11b^+^CD11c^-^. (**H-G**) In lymphnodes (dLNs) of *L*. *major* infected WT and CatB^-/-^ DCs were gated on (**G**) CD11c^+^ cells from myeloïd cells and FACS analysis of the percentage of (**H**) CD11c^+^CD11b^-^. Data depicted represent the mean and SEM of at least 3 independent experiments with n ≥ 4 mice/group/time point. * p<0.05;
(PDF)

S5 Fig Footpad iNOS transcripts and IgG2a/IgG1 antiobody ratio. (**A**) iNOS mRNAs expression was by RT PCR in footpads of WT and CatB^-/-^ mice after infection with *L*. *major*. (**B**) Ratio of seric IgG2a and IgG1 from WT and CatB^-/-^ mice infected with *L*. *major*
(PDF)

S6 Fig Cell composition in naive spleens of WT and CatB^-/-^ mice. Splenocytes from naive WT and Cat^-/-^ mice were stained for CD4, CD25 and CD8 before purification of CD3 cells for adoptive transfers. Representative data of 2 experiments with n = 4 mice per group. Values are shown as mean ± SEM.
(PDF)
