# Supplementary material for: Cathepsin B-Deficient Mice Resolve Leishmania major Inflammation Faster in a T Cell-Dependent Manner
Source: PLoS Negl Trop Dis. 2016 May 16;10(5):e0004716. doi: 10.1371/journal.pntd.0004716 (PMC4868322; doi:10.1371/journal.pntd.0004716)
Supplement: S1 Fig — WT, CatS-/-, CatL-/- and AEP-/- mice were subcutaneously inoculated with 3x10^6^ stationary phase promastigotes of L. major in the footpads and experimental read-outs were measured at indicated time-points. Logarithm base 10 of limit dilution for parasite burden from footpads of infected mice. Data are presented as mean+SD. n = 3–7 mice per group/time-point. (PDF) [file pntd.0004716.s001.pdf]

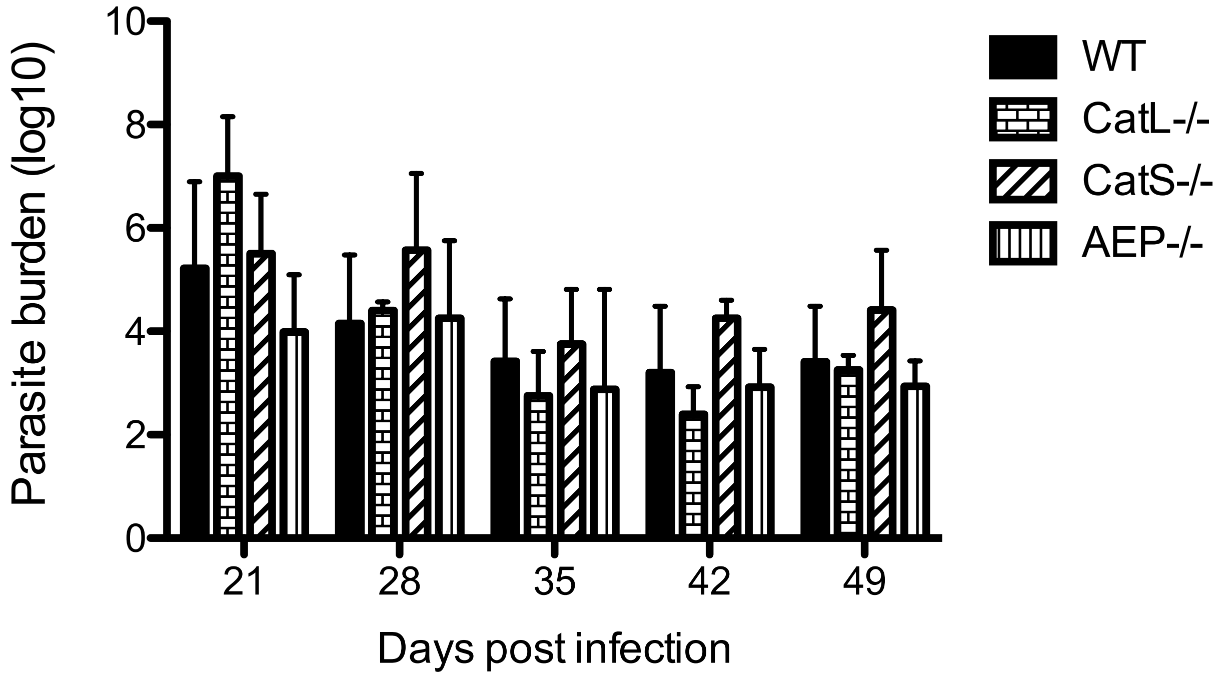

Supplementary Figure 1. Parasite burdens of WT, CatL-, CatS- and AEP-knockout mice

WT, CatS$^{-/-}$, CatL$^{-/-}$ and AEP$^{-/-}$ mice were subcutaneously inoculated with $3x10^6$ stationary phase promastigotes of *L. major* in the footpads and experimental read-outs were measured at indicated time-points. Logarithm base 10 of limit dilution for parasite burden from footpads of infected mice. Data are presented as mean+SD. n=3-7 mice per group/timepoint.
