# Supplementary material for: Cathepsin B-Deficient Mice Resolve Leishmania major Inflammation Faster in a T Cell-Dependent Manner
Source: PLoS Negl Trop Dis. 2016 May 16;10(5):e0004716. doi: 10.1371/journal.pntd.0004716 (PMC4868322; doi:10.1371/journal.pntd.0004716)
Supplement: S2 Fig — BMDMs from WT and CatB-/- mice were stimulated with LPS (100ng/ml), CpG (250ng /ml), live or killed L. major promastigotes (1:5 MOI) to determine TNFα production by ELISA (A) and to assess cytokine mRNAs expression by RT PCR (B and C). (PDF) [file pntd.0004716.s002.pdf]

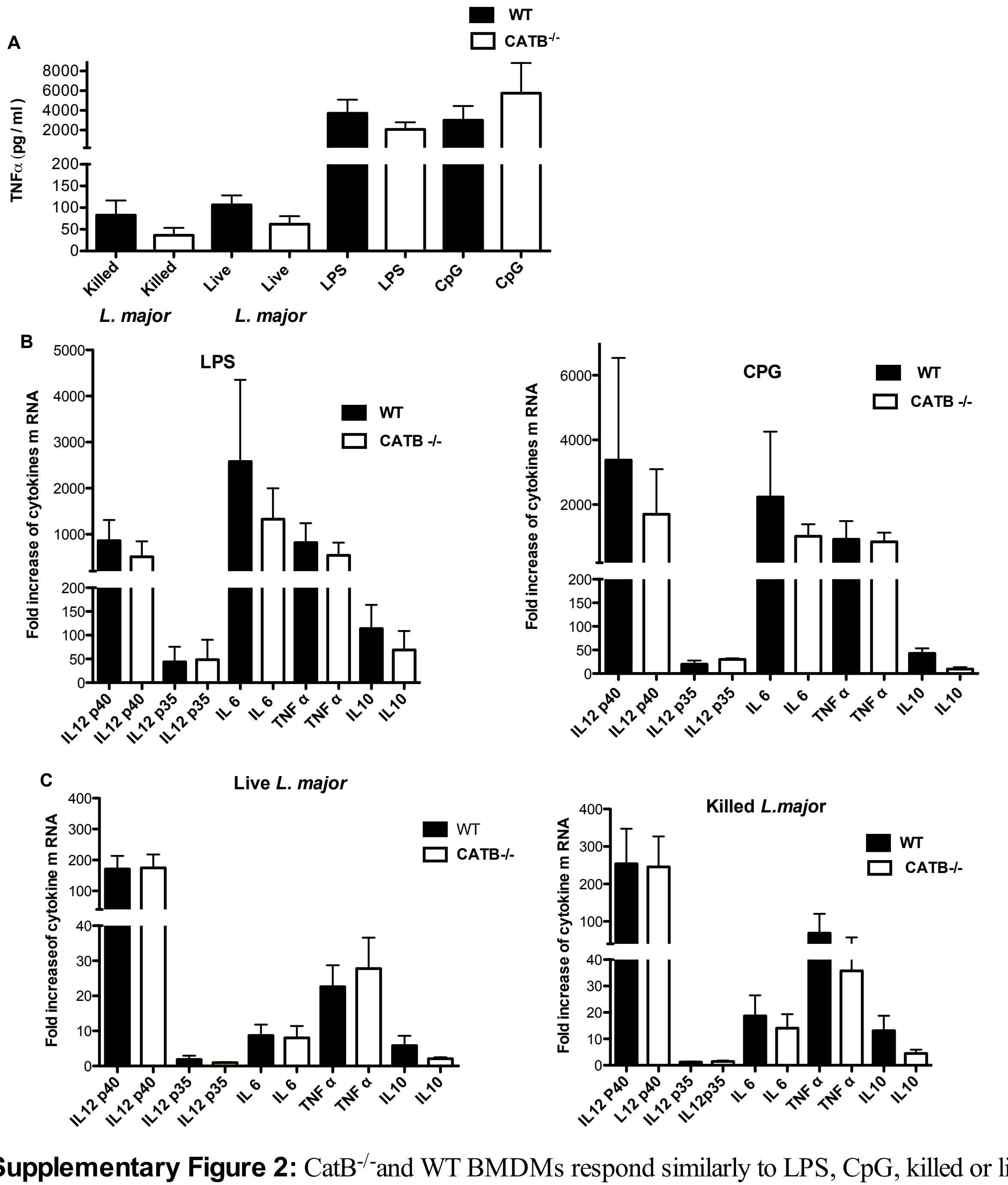

Supplementary Figure 2: $CatB^{-/-}$ and WT BMDMs respond similarly to LPS, CpG, killed or live *L. major*. BMDMs from WT and $CatB^{-/-}$ mice were stimulated with LPS (100ng/ml), CpG (250ng /ml), live or killed *L. major* promastigotes (1:5 MOI) to determine TNFα production by ELISA (**A**) and to assess cytokine mRNAs expression by RT PCR (**B** and **C**).
