# Supplementary material for: Cathepsin B-Deficient Mice Resolve Leishmania major Inflammation Faster in a T Cell-Dependent Manner
Source: PLoS Negl Trop Dis. 2016 May 16;10(5):e0004716. doi: 10.1371/journal.pntd.0004716 (PMC4868322; doi:10.1371/journal.pntd.0004716)
Supplement: S3 Fig — WT and CatB-/- were inoculated subcutaneously in the footpads with 3x10^6^ stationary phase promastigotes of L. major. Footpads were harvested at day 21 and 28 post infection. Levels of CXCL1 (A), CCL2 (B) and CCL5 (C) were measured in the supernatants of footpads by ELISA. (PDF) [file pntd.0004716.s003.pdf]

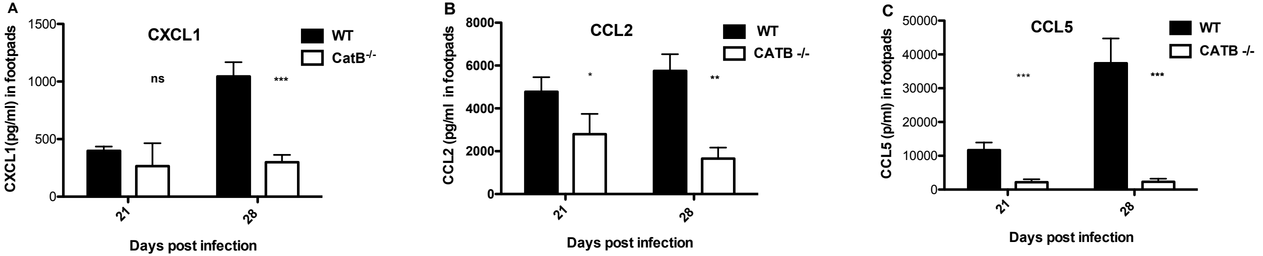

Supplementary Figure 3: Chemokines responses of *L. major* infected WT and CatB$^{-/-}$mice. WT and CatB-/- were inoculated subcutaneously in the footpads with $3x10^6$ stationary phase promastigotes of *L. major*. Footpads were harvested at day 21 and 28 post infection. Levels of CXCL1 **(A)** ,CCL2 **(B)** and CCL5 **(C)** were measured in the supernatants of footpads by ELISA.
