# Supplementary material for: Cathepsin B-Deficient Mice Resolve Leishmania major Inflammation Faster in a T Cell-Dependent Manner
Source: PLoS Negl Trop Dis. 2016 May 16;10(5):e0004716. doi: 10.1371/journal.pntd.0004716 (PMC4868322; doi:10.1371/journal.pntd.0004716)
Supplement: S4 Fig — Mice were harvested weekly as indicated. (A-C). Lymphnode samples prepared from L. major-infected WT (filled bars) and CatB-/- (empty bars) mice. CD11b+ CD11c- were gated on live events from lymphnodes single cell suspensions and FACS analysis of the percentage of (A) Ly6G-Ly6Chigh monocytes (B) Ly6G-Ly6Cint macrophages and (C) Ly6GhighLy6Cint neutrophils were performed, (D) NK were gated on lymphocytes. (E-F) In footpads (FPs) of L. major infected WT and CatB-/-, CD45+ were gated on live events for facs analysis of DCs (E) CD11b+CD11c+ and (F) CD11b+CD11c-. (H-G) In lymphnodes (dLNs) of L. major infected WT and CatB-/- DCs were gated on (G) CD11c+ cells from myeloïd cells and FACS analysis of the percentage of (H) CD11c+CD11b-. Data depicted represent the mean and SEM of at least 3 independent experiments with n ≥ 4 mice/group/time point. * p<0.05; (PDF) [file pntd.0004716.s004.pdf]

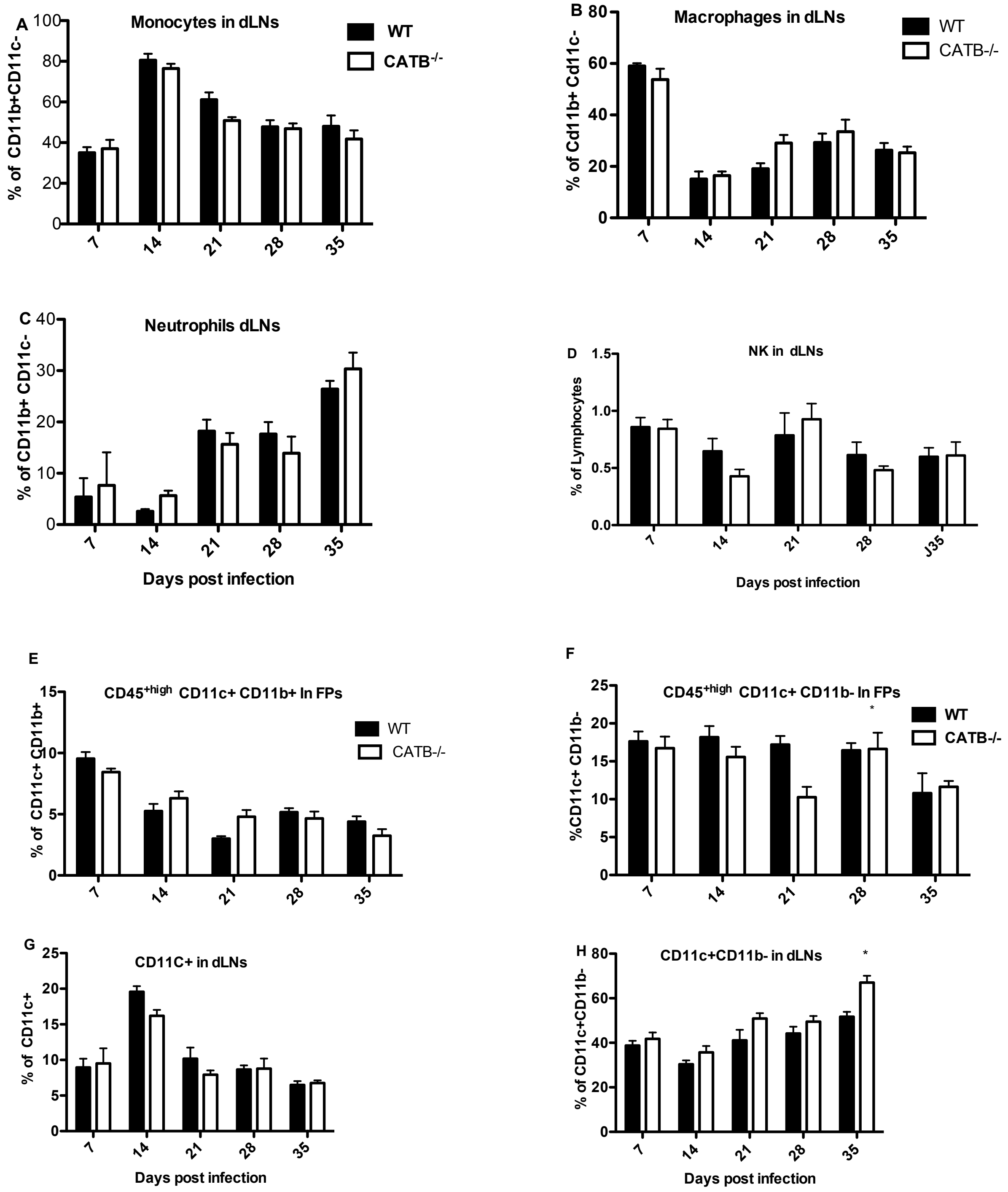

Supplementary Figure 4: Innate immune cells in lymphnodes (dLNs) or footpads (FPs) of *L. major* infected WT and CatB$^{-/-}$ mice which were harvested weekly as indicated. **(A-C)**. Lymphnodes samples prepared from *L. major* -infected WT (filled bars) and CatB$^{-/-}$ (empty bars) mice. CD11b+ CD11c- were gated on live events from lymphnodes single cell suspensions and FACS analysis of the percentage of **(A)** Ly6G^{-}Ly6C^{high} monocytes **(B)** Ly6G^{-}Ly6C^{int} macrophages and **(C)** Ly6G^{high}Ly6C^{int} neutrophils were performed, (D) NK were gated on lymphocytes. **(E-F)** In footpads (FPs) of *L. major* infected WT and CatB$^{-/-}$, CD45+ were gated on live events for facs analysis of DCs **(E)** CD11b^{+}CD11c^{+} and **(F)** CD11b^{+}CD11c^{-}**. (H-G)** In lymphnodes (dLNs) of *L. major* infected WT and CatB$^{-/-}$ DCs were gated on (G) CD11c^{+} cells from myeloïd cells and FACS analysis of the percentage of (H) CD11c+CD11b -Data depicted represent the mean and SEM of at least 3 independent experiments with n ≥ 4 mice/group/time point. * $p<0.05$;
