# Supplementary material for: Cathepsin B-Deficient Mice Resolve Leishmania major Inflammation Faster in a T Cell-Dependent Manner
Source: PLoS Negl Trop Dis. 2016 May 16;10(5):e0004716. doi: 10.1371/journal.pntd.0004716 (PMC4868322; doi:10.1371/journal.pntd.0004716)
Supplement: S5 Fig — (A) iNOS mRNAs expression was by RT PCR in footpads of WT and CatB-/- mice after infection with L. major. (B) Ratio of seric IgG2a and IgG1 from WT and CatB-/- mice infected with L. major (PDF) [file pntd.0004716.s005.pdf]

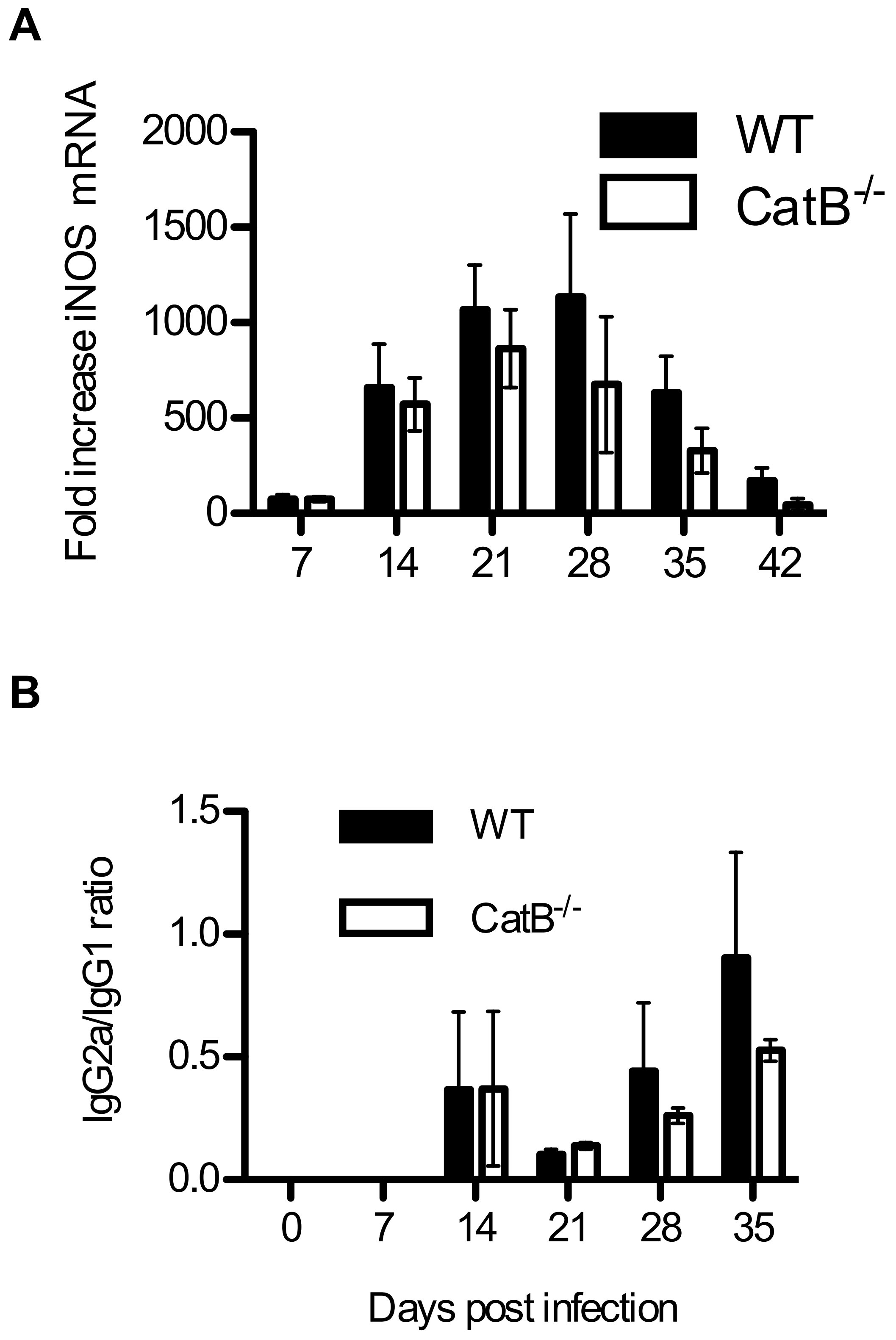

Supplementary Figure 5 - FP iNOS transcript and IgG2a/IgG1 antiobody ratio.
a) iNOS mRNAs expression was by RT PCR in footpads of WT and CatB mice after infection with *L. major*
b) Ratio of seric IgG2a and IgG1 from WT and CatB mice infected with *L. major*
