# Supplementary material for: Cathepsin B-Deficient Mice Resolve Leishmania major Inflammation Faster in a T Cell-Dependent Manner
Source: PLoS Negl Trop Dis. 2016 May 16;10(5):e0004716. doi: 10.1371/journal.pntd.0004716 (PMC4868322; doi:10.1371/journal.pntd.0004716)
Supplement: S6 Fig — Splenocytes from naive WT and Cat-/- mice were stained for CD4, CD25 and CD8 before purification of CD3 cells for adoptive transfers. Representative data of 2 experiments with n = 4 mice per group. Values are shown as mean ± SEM. (PDF) [file pntd.0004716.s006.pdf]

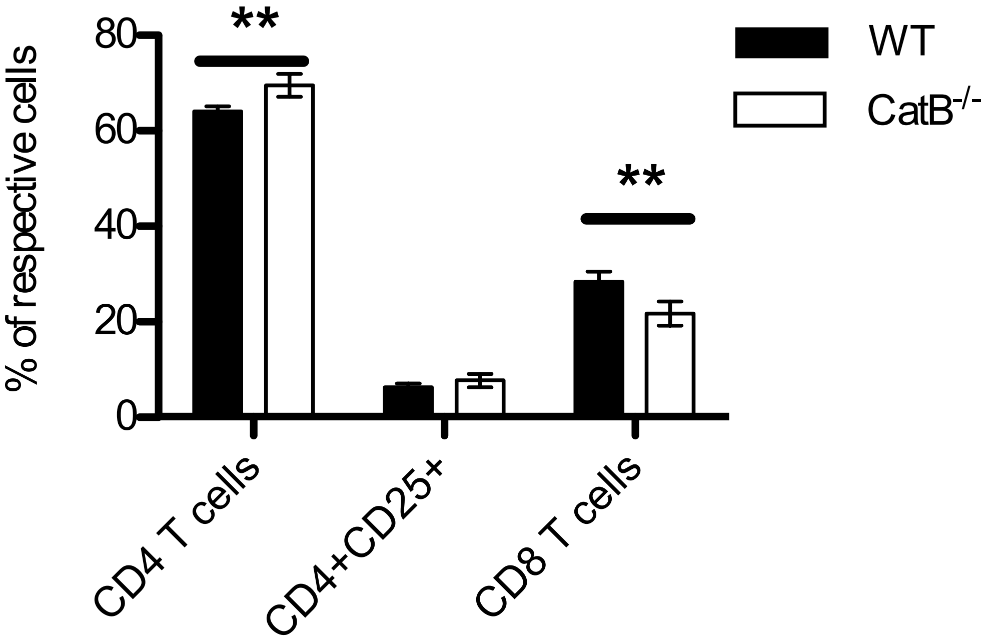

Supplementary Figure 6 - Cell composition in naive spleens of WT and CatB$^{-/-}$ mice.
Splenocytes from naive WT and Cat$^{-/-}$ mice were stained for CD4, CD25 and CD8 before purification of CD3 cells for adoptive transfers. Representative data of 2 experiments with n=4 mice per group. Values are shown as mean ± SEM.
